# A highly magnetized long-period radio transient exhibiting unusual emission features

**DOI:** 10.1126/sciadv.adp6351

**Published:** 2025-01-17

**Authors:** Yunpeng Men, Sam McSweeney, Natasha Hurley-Walker, Ewan Barr, Ben Stappers

**Affiliations:** ^1^ Max-Planck-Institut für Radioastronomie, Auf dem Hügel 69, Bonn, D-53121, Germany.; ^2^ International Centre for Radio Astronomy Research, Curtin University, 1 Turner Ave, Bentley, WA, 6102, Australia.; ^3^ Jodrell Bank Centre for Astrophysics, Department of Physics and Astronomy, The University of Manchester, Oxford Road, Manchester, M13 9PL, UK.

## Abstract

Long-period radio transients are a new class of astrophysical objects that exhibit periodic radio emission on timescales of tens of minutes. Their true nature remains unknown; possibilities include magnetic white dwarfs, binary systems, or long-period magnetars; the latter class is predicted to produce fast radio bursts (FRBs). Using the MeerKAT radio telescope, we conducted follow-up observations of the long-period radio transient GPM J1839-10. Here we report that the source exhibits a wide range of unusual emission properties, including polarization characteristics indicative of magnetospheric origin, linear-to-circular polarization conversion, and drifting substructures closely resembling those observed in repeating FRBs. These radio characteristics provide evidence in support of the long-period magnetar model and suggest a possible connection between long-period radio transients, magnetars, and FRBs.

## Teaser

A long-period radio transient exhibits unusual emission features that could potentially reveal its physical nature.

## INTRODUCTION

Long-period radio transients (LPRTs), a new identified class of astrophysical objects, exhibit periodic radio emissions on timescales of tens of minutes (*1*–*3*). These sources can manifest as either short-lived (*1*) or long-lived (*2*) characteristic, and their physical nature remains a subject of active debate (*4*, *5*). Proposed models include magnetized white dwarfs (*6*), binary systems (*7*), and long-period magnetars (*8*). Magnetars, characterized as young and highly magnetized neutron stars with higher magnetic field strengths than typical pulsars, ranging from 10^13^ to 10^15^ G (*9*), represent one of the suggested models. Now, six magnetars are known to exhibit radio emission with periods spanning from 2 to 12 s (*10*–*15*).

A wide range of similarities exist among the observed emission properties of normal pulsars, millisecond pulsars, and radio magnetars, despite differences in their magnetic field structures and magnetosphere sizes (*16*). These shared emission properties include quasi-periodicity, significant circular polarization (CP), orthogonal polarization modes (OPMs), and the association between the sign change of CP and rapid position angle (PA) swing. The suggestion of the long-period magnetar model proposes that these similar emission properties can also be extended to LPRTs.

Fast radio bursts (FRBs) are bright radio bursts lasting from microseconds to tens of milliseconds (*17*–*19*), and their physical origin and emission mechanism remain unknown. However, a Galactic FRB, FRB 200428, was identified producing from the radio magnetar SGR 1935+2154 (*15*, *20*), suggesting a potential association between magnetars and a subset of FRBs. Therefore, the radio magnetars could explain for both the LPRTs and FRBs. Furthermore, the hypothesis of a long-period magnetar, although not confirmed, has been proposed to elucidate certain FRB properties, including periodic activity (*21*). The validation of this potential connection remains a subject of ongoing investigation.

GPM J1839-10, discovered in the Murchison Widefield Array observation, has exhibited activity for three decades, featuring a period of 1318.1957 ± 0.0002 s (*2*). This establishes it as the longest-lived LPRT reported to date. Previous observations revealed that GPM J1839-10 has a dispersion measure (DM) of 273.5 ± 2.5 cm^−3^ pc and a rotation measure (RM) of 531.63 ± 0.15 rad m^−2^ (*2*). The period derivative constraint of $\dot{P}≲3.6\times 10^{-13}\;s\;s^{-1}$ was derived from archival data of GPM J1839-10. The combined characteristics of a slow period and a low period derivative place GPM J1839-10 at the very edge of the most generous death line (*2*), which is established based on the rotating dipolar magnetic fields and pair production mechanisms that typically account for coherent emission from normal pulsars (*22*).

## RESULTS AND DISCUSSION

### Radio observation and pulse morphology

**Fig. 1.**
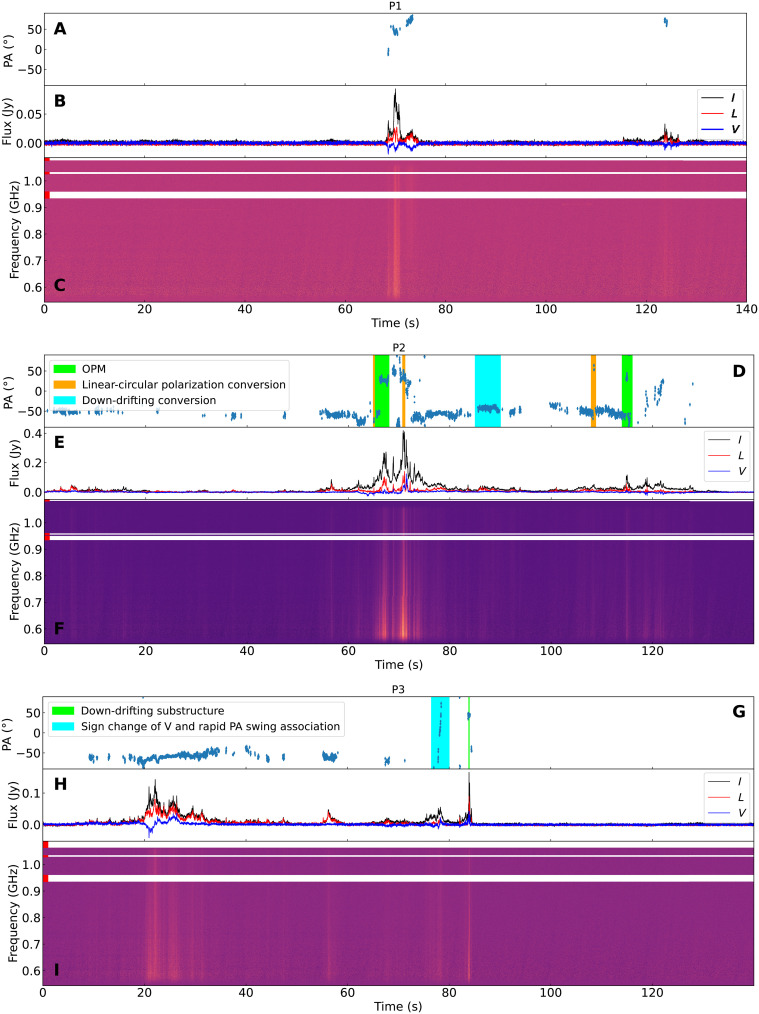
Dynamic spectra aligned in phase of the three pulses detected on 20 August 2023. (**A**, **D**, and **G**) Time evolution of the PA. (**B**, **E**, and **H**) Temporal variations of total intensity (*I*, black), linear polarization (LP) intensity (*L*, red), and CP intensity (*V*, blue). (**C**, **F**, and **I**) Dynamic spectrum of total intensity corrected for dispersion at 274.12 ± 0.25 cm^−3^ pc. The Faraday correction was applied using RMs of 531.05 ± 0.02, 530.76 ± 0.02, and 530.76 ± 0.01 rad m^−2^ for each pulse, respectively. The frequency channels affected by RFI contamination are mitigated and indicated by white strips on the dynamic spectrum (see Materials and Methods).

A 2.5-hour follow-up polarimetric observation for GPM J1839-10 was conducted with MeerKAT on 20 August 2023 [universal time (UT)]. The observation was performed in the UHF band, covering frequencies from 544 to 1088 MHz, with a time resolution of 15 μs. We detected pulsed emissions in three periods, denoted as P1, P2, and P3 in the following text (Fig. 1), which gives a rough event rate of pulsed emission from GPM J1839-10 of 20%. The DMs for the three pulses are 274.5 ± 1.9, 274.12 ± 0.25, and 274.0 ± 0.9 cm^−3^ pc, which are consistent with the earlier observation on 20 July 2022 (UT) where the DM was measured to be 273.5 ± 2.5 cm^−3^ pc (*2*). The narrowest burst component has a width of about 10 ms. The pulsed emission reaches a peak flux density ranging between 0.1 and 0.4 jansky (Jy) in the UHF band, resulting in an isotropic luminosity of $2.1_{-1.5}^{+2.8}\times 10^{30}$ to $8.5_{-6.0}^{+10.8}\times 10^{30}\;\text{erg}\;s^{-1}$ (see Materials and Methods).

These pulses last for about 80 to 120 s, exhibiting complex morphologies, despite similarities in the phases with radio emission, accounting for roughly 10% of the period. The P2 pulse exhibits quasi-periodic emission structures with a quasi-period of 1.97 s at a significance level of 3.6-σ (see Materials and Methods). Quasi-periodic emission structures have been identified in normal radio pulsars (*23*, *24*), rotating radio transients (*25*), and radio magnetars (*26*). An empirical relationship between the rotational period and the period of quasi-periodic structures has been established for these sources (*26*). This relationship can be extended to the quasi-periodic structures of the P2 pulse detected in this observation as well (fig. S1).

### Down-drifting substructure

**Fig. 2.**
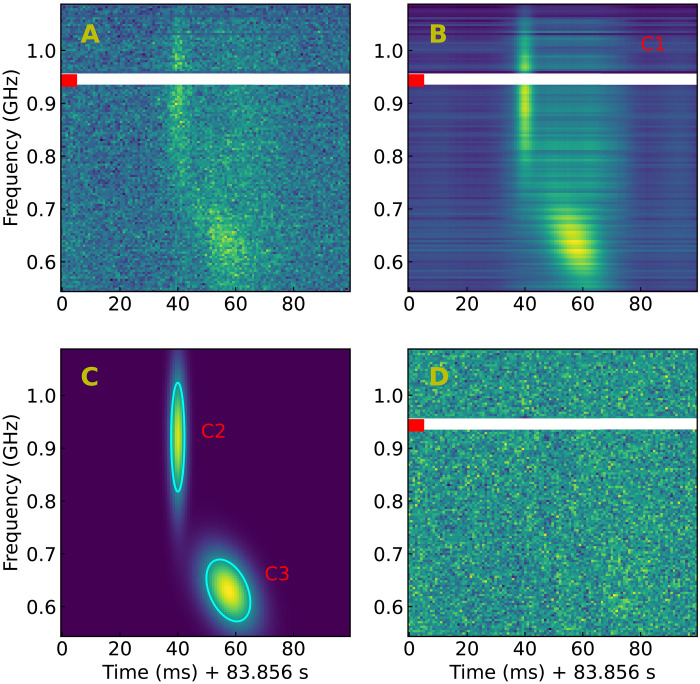
Spectrum fitting of the down-drifting substructure within the P3 pulse. (**A**) Dynamic spectrum. (**B**) Fitted dynamic spectrum showcasing the broadband emission C1 with the two narrowband emission components C2 and C3. (**C**) Fitted dynamic spectrum depicting the emission components C2 and C3. The cyan elliptical circle denotes the FWHM width of the fitted 2D Gaussian shape (see Materials and Methods). (**D**) Residual dynamic spectrum. The time is aligned with the P3 pulse in Fig. 1.

With the high time resolution observation of MeerKAT, we discovered that the short-duration substructures display drifting behaviors within the dynamic spectrum of GPM J1839-10. In the P3 pulse, a substructure demonstrating multiple emission components displays both single-component down-drifting and multicomponent down-drifting characteristics (Fig. 2). The substructure comprises a wideband emission component (C1) along with two narrowband emission components (C2 and C3). The two narrowband emission components can be fitted using a two-dimensional (2D) Gaussian shape (see Materials and Methods). The emission component C2 exhibits no apparent frequency drifting, featuring a full width at half maximum (FWHM) bandwidth of 206 ± 7 MHz and a FWHM width of 4.6 ± 0.2 ms. Conversely, the emission component C3 displays a FWHM bandwidth of 117 ± 4 MHz, a FWHM width of 15.2 ± 0.5 ms, and a single-component down-drifting rate of −5.3 ± 0.5 MHz ms^−1^. There is a central time offset of 17.4 ± 0.2 ms between the two emission components C2 and C3. Their respective center frequencies are 920 ± 3 and 630 ± 2 MHz, showcasing a multicomponent down-drifting rate of −16.6 ± 0.2 MHz ms^−1^.

Frequency drifting behavior has been observed in FRBs (*27*–*31*) and solar bursts (*32*). The frequency drifting behavior observed in solar bursts typically involves longer timescales and slower frequency drifting rates compared to this substructure of GPM J1839-10. For instance, solar type III radio bursts usually exhibit a higher-frequency drifting rate, around 0.1 MHz ms^−1^, and a duration of a few seconds (*33*). In contrast, repeating FRBs showcase diverse features, including multicomponent down-drifting (also known as the “sad trombone effect”) and single-component down-drifting (*34*–*36*). The frequency drifting rates in these events can vary from a few megahertz per millisecond to several thousand megahertz per millisecond (*28*, *29*, *31*, *35*–*37*). The drifting rate observed in this substructure of GPM J1839-10 is akin to the drifting rates witnessed in repeating bursts of the Canadian Hydrogen Intensity Mapping Experiment (CHIME) observed within a similar frequency band (*28*, *31*). In addition, bursts from repeating FRBs exhibit narrowband emission features, with bandwidths ranging from approximately 50 to 400 MHz within the 400- to 800-MHz band (*38*). These durations of these bursts typically span from a few milliseconds to tens of milliseconds (*38*). Both the bandwidth and duration of the emission components in this substructure of GPM J1839-10 fall within the range observed in CHIME repeating FRBs (fig. S2). This similarity in frequency drifting behavior suggests a shared emission mechanism or propagation effect between GPM J1839-10 and repeating FRBs (*39*–*42*).

### Polarization properties

Using the MeerKAT full-Stokes data, we measured the RMs of the three pulses, which are 530.68 ± 0.05, 530.33 ± 0.05, and 530.44 ± 0.04 rad m^−2^, respectively (see Materials and Methods). The RM variation among these three pulses is less than 1 rad m^−2^. In addition, the RM change from the previous observation on 20 July 2022 (UT) is also less than 2 rad m^−2^, with an RM value of 531.63 ± 0.15 rad m^−2^ (fig. S3). Upon correction for Faraday rotation using the measured RMs, the polarized profile of the three pulses was derived. The polarization characteristics exhibit complex variations across the observed period. The linear polarization (LP) fraction shows fluctuations ranging from approximately 0 to 100% across different phases, whereas the CP fraction reaches values as high as about 95%, exhibiting an association between the sign change and rapid PA swing at specific phases (fig. S4). Furthermore, the PA swing exhibits complex variations with phase, being relatively constant in some phase ranges while displaying steep changes in others (Fig. 1), and OPMs are detected in the PA swing (fig. S5).

The PA swing and occurrence of OPMs are frequently observed in the polarization emission of pulsars. The PA swing is attributed to variation in the direction of the magnetic field as the line of sight crosses the emission region (*43*). In addition, the association between the rapid PA swing and the sign change of CP was suggested to be caused by the geometry effect when the line of sight sweeps the emission cones in a pulsar magnetosphere (*44*). Furthermore, while the PA swing in pulsars is often explained by the rotating vector model (*43*), this model does not apply straightforwardly to GPM J1839-10, as it exhibits multiple irregular PA changes within approximately ^1^/_10_ of its period (Fig. 1). This discrepancy indicates that the emission region of GPM J1839-10 has a complex magnetic field configuration similar to radio magnetars (*45*–*47*). The PA swings can be explained by magnetospheric models as opposed to the synchrotron maser mechanism because the latter predicts a constant PA profile for the emitted radiation due to the requirement of ordered magnetic fields in the shocked region where the emission takes place. The observed PA swing profiles in GPM J1839-10, therefore, favor a magnetospheric origin. It should be noted that diverse PA swings were also observed for FRBs, and a similar interpretation was drawn as well (*48*). Moreover, the emission of GPM J1839-10 exhibits significant CP and LP fractions in certain phases, akin to the polarization properties observed in normal pulsars (*49*), radio magnetars (*46*), and FRBs (*50*, *51*). This similarity implies the presence of common emission mechanisms or propagation effects among them.

### Linear-to-circular polarization conversion

**Fig. 3.**
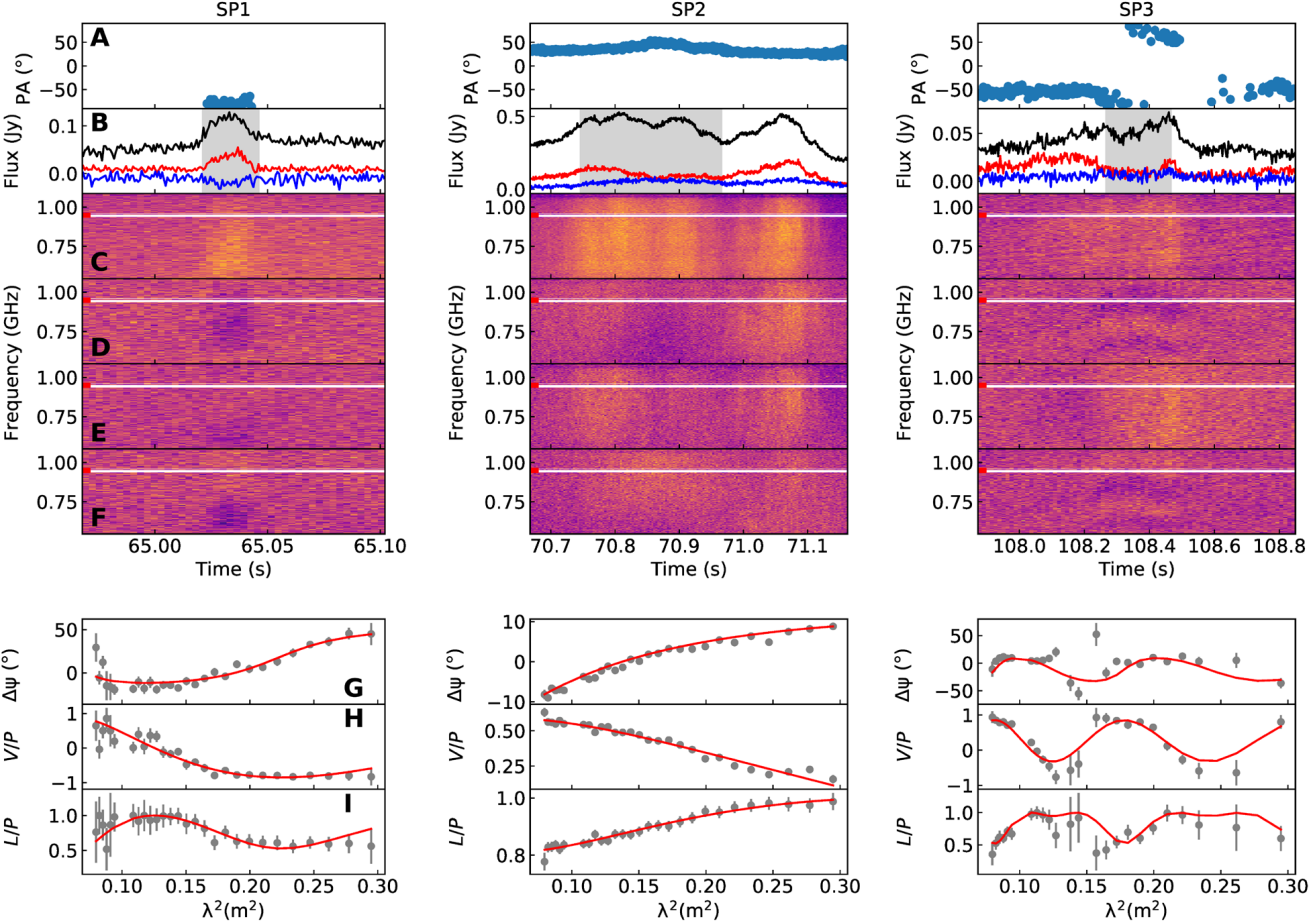
Stokes spectra of the subpulses, exhibiting linear-to-circular polarization conversion within the P2 pulse. (**A**) PA variation. (**B**) Total intensity (*I*, black), LP intensity (*L*, red), and CP intensity (*V*, blue). (**C**) Stokes *I* spectrum. (**D**) Stokes *Q* spectrum. (**E**) Stokes *U* spectrum. (**F**) Stokes *V* spectrum. (**G**) Faraday conversion fitting of the PA residuals after the Faraday rotation correction (see Materials and Methods). (**H**) Faraday conversion fitting of the normalized CP residuals. (**I**) Faraday conversion fitting of the normalized LP residuals.

Within the subpulses of P2, there are evident instances of frequency-dependent conversion between linear and circular polarized emission (Fig. 3). To investigate this behavior, we focused on three subpulses (referred to as SP1, SP2, and SP3 hereafter) within P2, demonstrating considerable frequency-dependent polarization variation. Notably, the polarization behavior varies across different phases of the pulse. Therefore, we extracted the PA residuals and LP and CP fractions within the specified phase range exhibiting stable frequency-dependent polarization variations (see Materials and Methods). SP1, characterized by its short duration, exhibits a sign change in the CP spectrum. Moreover, both the LP fraction and PA residuals showcase frequency-dependent variations. SP2 falls within the phase range marked by the peak flux of P2, demonstrating a noticeable decrease in CP fraction and an increase in LP fraction. Similarly, its PAs exhibit frequency-dependent residuals. In SP3, oscillations are observed in both CP and LP, alongside rapid variations in PA concerning the pulse phase. To characterize the polarization variation, we used a phenomenological model (*52*) to fit the spectrum (see Materials and Methods). The generalized rotation measures (GRMs) of SP1, SP2, and SP3 are $20.01_{-8.8}^{+0.45}$, $4.16_{-0.49}^{+0.44}$, and $45.97_{-0.6}^{+1.0}$ with corresponding wavelength-dependent indices of $0.35_{-0.24}^{+0.39}$, $0.84_{-0.16}^{+0.23}$, and $0.95_{-0.13}^{+0.17}$.

The linear-to-circular polarization conversion was also observed in FRB 20201124A (*50*) and the radio-loud magnetar XTE J1810-197 (*53*). FRB 20201124A was reported to exhibit linear-to-circular polarization conversion with wavelength-dependent indices of 2 (*50*) and 3 (*54*). In contrast, the magnetar XTE J1810-197, which shows linear-to-circular polarization conversion with a wavelength-dependent index ranging from 0 to 3 (*53*), is more similar to GPM J1839-10. Furthermore, the limited RM variation indicates the near-field origin of its polarization conversion behavior, which can be produced in the magnetosphere. These findings support the presence of a similar local magneto-ionic environment around GPM J1839-10 and XTE J1810-197. Given that Faraday conversion typically occurs in strong magnetic fields (*55*), we can potentially estimate the local magnetic field strength where the polarization conversions take place, $B∼\frac{300}{\gamma }\;G$ (see Supplementary Text).

### Down-drifting polarization conversion

**Fig. 4.**
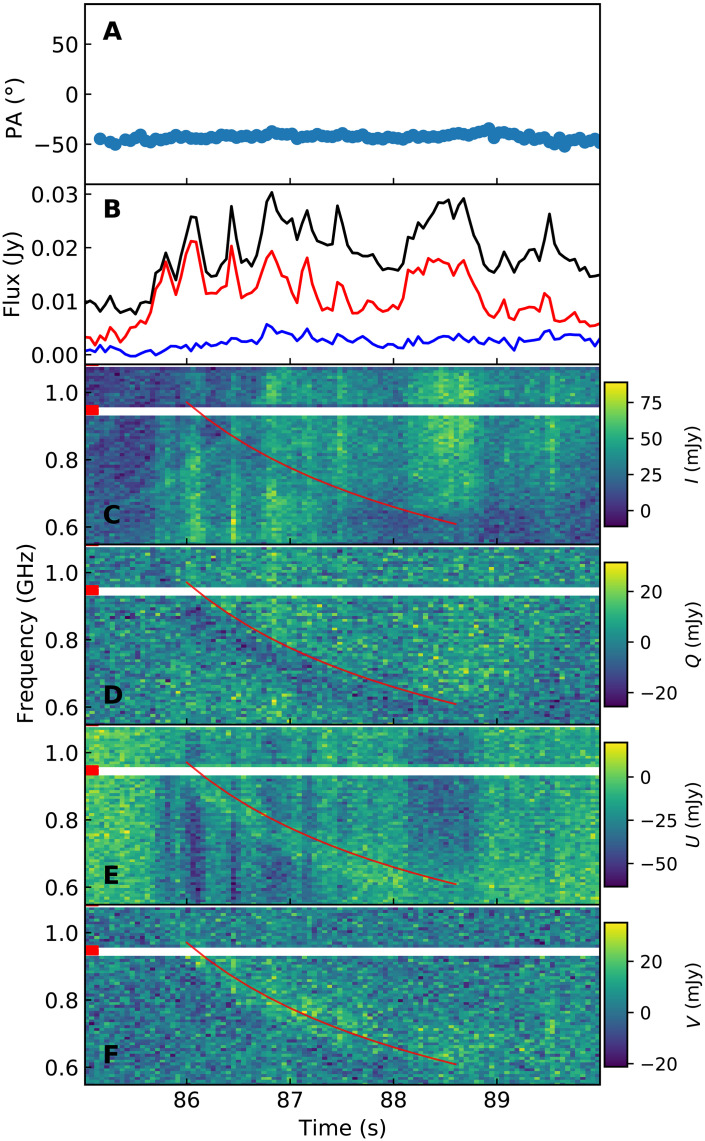
Stokes spectra of the substructure with down-drifting polarization conversion within the P2 pulse. (**A**) PA variation. (**B**) Total intensity (*I*, black), LP intensity (*L*, red), and CP intensity (*V*, blue). (**C**) Stokes *I* spectrum. (**D**) Stokes *Q* spectrum. (**E**) Stokes *U* spectrum. (**F**) Stokes *V* spectrum. The red lines in the spectra represent the fitted frequency-dependent relation of the down-drifting conversion (see Materials and Methods).

In the P2 pulse, we observed a statistically significant down-drifting polarization conversion behavior in the Stokes spectra (Fig. 4). A distinct down-drifting band exhibited prominent linear-to-circular polarization conversion. Outside this band, the emission displayed a small CP fraction, while within the band, CP emission intensified. Moreover, the total intensity decreased by an average of about 50% in the down-drifting band. The drift rate was $4.2_{-0.1}^{+0.1}$ MHz s^−1^ with a frequency relation index of $1.95_{-0.61}^{+0.03}$ (see Materials and Methods). This radio emission characteristic has not been reported previously. The current theoretical models for down-drifting structures (*39*–*42*) and polarization conversion behaviors (*55*) face challenges in explaining the observed concurrence of the two emission characteristics. This presents an unprecedented opportunity to investigate both down-drifting and polarization conversion mechanisms.

### Implications

The physical nature of LPRTs remains mysterious (*1*, *2*), with various models proposed, such as magnetized white dwarfs (*6*), binary systems (*7*), and long-period magnetars (*8*). In this study, we present the discovery of diverse emission properties in GPM J1839-10 that resemble those observed in radio magnetars or pulsars, including quasi-periodicity, OPMs, significant CP and LP, and the association between the sign change of CP and rapid PA swing, which are produced from their magnetospheres. These observed similar radio emission properties provide evidence for the magnetospheric origin of the radio emission of GPM J1839-10. Furthermore, GPM J1839-10 exhibits a similar behavior of linear-to-circular polarization conversion as the magnetar XTE J1810-197, which indicates a similar magnetosphere environment around GPM J1839-10 with radio magnetars, supporting the long-period magnetar model. In addition, a down-drifting substructure observed in GPM J1839-10 resembles the repeating FRBs. The observed similarities in emission properties in our observation suggest a possible connection between LPRTs, magnetars, and FRBs.

## MATERIALS AND METHODS

### Radio observations

MeerKAT, operated by the South African Radio Astronomy Observatory, is a large radio interferometer comprising 64 dishes, each 13.5 m in size, situated in the Northern Cape of South Africa (*56*). The follow-up observation of GPM J1839-10 was conducted under the project code DDT-20220718-NH-01 in the UHF band, spanning a frequency range of 544 to 1088 MHz. The observations comprised 15 sessions, each lasting approximately 10 min, equivalent to half of the period of GPM J1839-10. This observation scheme allows for the complete coverage of 15 periods within a total observation time of 2.5 hours (table S1). The full polarization data were recorded using the pulsar timing user-supplied equipment (PTUSE) backend (*57*), operating in the PSRFITS search mode, using 1024 frequency channels, and a time resolution of 15 μs. Notably, the recorded data have already undergone polarization calibration (*58*). We processed the data using DSPSR (*59*) to generate archive files containing the full polarization spectrum with a time resolution of 12 ms post-downsampling. Consequently, only last three periods exhibited detectable radio emission. For the remaining periods, we conducted a single pulse search using TransientX (*60*), using a pulse width search range below 100 ms, a DM range of 250 to 300 cm^−3^ pc, and a signal-to-noise ratio (S/N ratio) threshold of 8. No radio pulses were detected in this search. Subsequently, we conducted DM estimation for the three identified pulses using DM_PHASE (*61*). This method extracts DMs by maximizing the pulse structures. The DMs obtained for the three pulses are 274.5 ± 1.9, 274.12 ± 0.25, and 274.0 ± 0.9 cm^−3^ pc, respectively. The RMs for the three pulses were obtained by fitting the Stokes Q and U spectrum (fig. S6), with a detailed description of the algorithm available in (*48*). The derived RMs for the three pulses are 531.05 ± 0.02, 530.67 ± 0.02, and 530.76 ± 0.01 rad m^−2^, respectively. It should be noted that we corrected the sign of the RM for the observation on 20 July 2022 (*2*) because the receiver handedness was not corrected. To account for the ionosphere’s RM contribution, the code ionFR (*62*) was applied. This correction resulted in revised RMs of 530.68 ± 0.05, 530.33 ± 0.05, and 530.44 ± 0.04 rad m^−2^ for the three pulses. Notably, there were no significant changes observed in either DM or RM compared to the observation conducted on 20 July 2022 (fig. S3).

### Radio luminosity

The flux density of the pulses were estimated using the radiometer equation

$$S=S/N\times \frac{T_{\text{sys}}}{G\;\sqrt{n_{p}\;\text{BW}\;T_{s}}}$$ (1)

where the S/N ratio is estimated by the ratio of the intensity of a sample to noise. The system temperature $T_{\text{sys}}$ is about 18 K, and the total antenna gain *G* is about 2.8 K/Jy (*57*). $n_{p}$ is 2, which is the number of polarization summed. The bandwidth BW is 544 MHz, while the time resolution is 12 ms. To estimate the luminosity, we use the DM distance of 5.7 ± 2.9 kpc estimated with the YMW16 model (*2*, *63*). The derived peak isotropic luminosities $L=4\pi \;d_{\text{DM}}^{2}\;S$ are $2.1_{-1.5}^{+2.8}\times 10^{30}$, $8.5_{-6.0}^{+10.8}\times 10^{30}$, and $3.5_{-2.7}^{+4.5}\times 10^{30}$ erg s^−1^.

### Quasi-periodicity

The P2 pulse exhibits multiple peak structures in its profile. To investigate the quasi-periodicity, we conducted the periodicity analysis using the autocorrelation function (ACF) and power spectral density (PSD). The ACF was computed by calculating the autocorrelation coefficient (ρ) of the total intensity (*I*) within the time range of 60-80 s, determined as

$$\rho \left(n\;T_{s}\right)=\frac{\sum_{i}I\left[t_{i}\right]\cdot I\left[t_{i}+n\;T_{s}\right]}{\sum_{i}I\left[t_{i}\right]\cdot I\left[t_{i}\right]}$$ (2)

where *T*_*s*_ is the time resolution of the intensity profile, and the time lag is $n\;T_{s}$. The PSD was derived from the same total intensity series using the SCIPY.SIGNAL.PERIODOGRAM. A quasi-period of 1.97 s is revealed in the ACF and PSD plots (fig. S7). We choose the quasi-period 1.97 s rather than the double period based on the intensity profile peaks. To assess the significance of this quasi-periodicity, we applied Fisher’s *g* test based on the PSD (*64*). As the test is conducted at the maximum PSD peak, the upper bound of the *P* value for the quasi-period was estimated of $P<2.7\times 10^{-4}$, equivalent to 3.6σ. We roughly measured the error of the quasi-period based on the width of the quasi-periodic structures of ∼1 s, which gives a geometric SD factor of 1.5 (*26*). We extracted the data in (*26*) and added the rotational period and period of quasi-periodic structures GPM J1839-10 to the relation, which can be fit in 2σ (fig. S1).

### Down-drifting structure

In the pulse of the period P3, we found a substructure exhibiting frequency down-drifting behavior (Fig. 2). To investigate the drifting substructure, we segmented a 100-ms data segment around it, using a time resolution of 0.2 ms using DSPSR. We performed dedispersion and Faraday rotation correction using the DM of 274 cm^−3^ pc and the RM of 530.76 rad m^−2^. To mitigate radio frequency interference (RFI), we manually identified and removed contaminated frequency channels using PAZI in PSRCHIVE. The data were then normalized to achieve unit variance and a zero offset in each frequency channel for subsequent analysis, using the off-pulse data. The substructure comprises three emission components: a broadband emission component (C1) with a wide profile and two narrowband components of shorter durations in the upper (C2) and lower bands (C3), respectively. To investigate the down-drifting structure, we characterized the total intensity spectrum *I* using a model given by

$$\begin{array}{c} I_{j,i}=I_{0,j,i}+I_{1,j,i}+I_{2,j,i},\\ I_{0,j,i}=c_{j}s_{i}+d_{j},\\ I_{1,j,i}=f\left(x_{i},y_{j},K_{1},x_{c,1},y_{c,1},a_{1},b_{1},\theta_{1}\right),\\ I_{2,j,i}=f\left(x_{i},y_{j},K_{2},x_{c,2},y_{c,2},a_{2},b_{2},\theta_{2}\right),\\ x_{i}=\frac{t_{i}}{0.1\;s},\\ y_{i}=\frac{f_{i}}{1\;\text{GHz}} \end{array}$$

where $I_{0}$, $I_{1}$, and $I_{2}$ represent the total intensity of emission components C1, C2, and C3. $c_{j}$ and $d_{j}$ stand for the amplitude and baseline offset of the common profile template *s* in the *j*th frequency channel of C1, respectively. In addition, $t_{i}$ and $f_{j}$ denote time and frequency, respectively. The function *f* represents a 2D Gaussian shape

$$\begin{array}{c} f\left(x,y,K,x_{c},y_{c},a,b,\theta \right)=K\text{exp}\left[-\left(\frac{{\text{cos}}^{2}\theta }{a^{2}}+\frac{{\text{sin}}^{2}\theta }{b^{2}}\right){\left(x-x_{c}\right)}^{2}\right.\\ -2\left(\frac{\text{sin}\theta \text{cos}\theta }{a^{2}}+\frac{\text{sin}\theta \text{cos}\theta }{b^{2}}\right)\left(x-x_{c}\right)\left(y-y_{c}\right)\\ \left.-\left(\frac{{\text{sin}}^{2}\theta }{a^{2}}+\frac{{\text{cos}}^{2}\theta }{b^{2}}\right){\left(y-y_{c}\right)}^{2}\right] \end{array}$$

where *K* denotes the peak intensity, and θ represents the declination angle. $x_{c}$ and $y_{c}$ denote the rescaled central time and frequency, respectively. *a* and *b* signify the rescaled duration and bandwidth. The FWHM bandwidth $B_{\text{FWHM}}$ and duration $W_{\text{FWHM}}$ of the emission component using the 2D Gaussian shape parameters are expressed as

$$B_{\text{FWHM}}=2\sqrt{\text{log}2}B\times 1\;\text{GHz}$$

$$W_{\text{FWHM}}=2\sqrt{\text{log}2}W\times 0.1\;s$$

$$B=\sqrt{\frac{4p}{4\mathrm{pq}-r^{2}}}$$

$$W=\sqrt{\frac{4q}{4\mathrm{pq}-r^{2}}}$$

$$p=\frac{{\text{cos}}^{2}\theta }{a^{2}}+\frac{{\text{sin}}^{2}\theta }{b^{2}}$$

$$q=\frac{{\text{sin}}^{2}\theta }{a^{2}}+\frac{{\text{cos}}^{2}\theta }{b^{2}}$$

$$r=2\text{sin}\theta \text{cos}\theta \left(\frac{1}{b^{2}}-\frac{1}{a^{2}}\right)$$

which represent the marginalized width along the time and frequency dimension, respectively. The single-component down-drifting rate $D_{s}$ is

$$D_{s}=\frac{\text{cos}\theta }{\text{sin}\theta }\times 10\;\text{MHz}\;s^{-1}$$ (3)

and the multicomponent down-drifting rate $D_{m}$ is calculated by dividing the center frequency and time offset of the emission components C2 and C3

$$D_{m}=\frac{y_{c,2}-y_{c,1}}{x_{c,2}-x_{c,1}}\times 10\;\text{MHz}\;s^{-1}$$ (4)

To estimate the profile *s* of the emission component C1, we extract the average profile from the spectrum within the frequency range of 720 to 730 MHz. This range is selected to minimize the signal contribution from emission components C2 and C3. The obtained average profile was subsequently smoothed using the Savitzky-Golay filter (*65*) to create the template *s*. To estimate the parameters within the total intensity spectrum model, we used the sampler MULTINEST (*66*), which relies on the nested sampling algorithm (*67*). Uniform priors were assigned to all parameters, and the resultant posterior distributions are depicted in fig. S8. The emission component C2 exhibits a derived FWHM bandwidth of 206 ± 7 MHz and a duration of 4.6 ± 0.2 ms, while the emission component C3 displays a FWHM bandwidth of 117 ± 4 MHz and a duration of 15.2 ± 0.5 ms. The single-component down-drifting rate of emission component C3 is measured at −5.3 ± 0.5 MHz ms^−1^, whereas the multicomponent down-drifting rate observed for C2 and C3 stands at −16.6 ± 0.2 MHz ms^−1^.

### Linear-to-circular polarization conversion

Within the P2 pulse, multiple subpulses display noticeable frequency-dependent variations in the full-Stokes spectrum. Among them, one subpulse demonstrates oscillations in polarization that vary with frequency. To investigate this polarization behavior, we focused on the data around three subpulses (SP1, SP2, and SP3) for further analysis. The data were initially normalized using the off-pulse range data, and we manually masked the frequency channels contaminated by RFI signals. The dispersion and Faraday rotation were corrected with the DM of 274.12 cm^−3^ pc and the RM of 530.67 rad m^−2^ estimated using the entire pulse of P2. We derived the frequency-dependent PA residual $\Delta \psi_{j}$, from the Stokes Q and U spectra, while correcting the time-dependent PA based on the equation

$${\hat{Q}}_{j}=\sum_{i}\left[Q_{j,i}\text{cos}\left(2\psi_{i}\right)+U_{j,i}\text{sin}\left(2\psi_{i}\right)\right]$$

$${\hat{U}}_{j}=\sum_{i}\left[U_{j,i}\text{cos}\left(2\psi_{i}\right)-Q_{j,i}\text{sin}\left(2\psi_{i}\right)\right]$$

$${\hat{L}}_{j}=\sqrt{{\hat{Q}}_{j}^{2}+{\hat{U}}_{j}^{2}}$$

$${\hat{P}}_{j}=\sqrt{{\hat{Q}}_{j}^{2}+{\hat{U}}_{j}^{2}+{\hat{V}}_{j}^{2}}$$

$$\Delta \psi_{j}=\frac{1}{2}\text{arctan}\frac{{\hat{U}}_{j}}{{\hat{Q}}_{j}}-\text{RM}\;\lambda_{j}^{2}$$

where $\psi_{i}$ represents the PA of the *i*th time sample, while $\lambda_{j}$ signifies the wavelength of the *j*th frequency channel. In addition, we derived the frequency-dependent LP and CP fractions, incorporating the generalized Weisberg correction in (*43*, *51*). These processes are conducted solely within time ranges, showing consistent and stable residuals across all three subpulses due to the varying nature of the polarization characteristics over time. Moreover, we enhanced the estimation of the PA residual and polarization fraction by aggregating values from neighboring frequency channels. The residual Stokes spectrum is depicted in Fig. 3, and the polarization vector variation on Poincaré sphere is shown in fig. S9, illustrating the linear-to-circular polarization conversion. Notably, SP3 exhibits frequency-dependent oscillations in the polarization fraction and PA residual. To characterize this polarization behavior, we applied the phenomenological model proposed in (*53*). In our study, we used a distinct representation, formulated as

$$p_{j}=R\left(u,\phi_{j}\right)\cdot p_{0}$$

where $p_{j}$ represents the observed unit polarization vector normalized by the total polarization intensity

$$p_{j}=\frac{1}{P_{j}}\left[\begin{array}{c} Q_{j}\\ U_{j}\\ V_{j} \end{array}\right]$$

$R\left(u,\phi_{j}\right)$ denotes the 3D rotation matrix with the polarization rotation axis u and the rotation angle $\phi_{j}$ in the jth frequency channel. The matrix R(u,ϕ) is defined as

$$R\left(u,\phi \right)=\left[\begin{array}{ccc} \text{cos}\phi +u_{x}^{2}\left(1-\text{cos}\phi \right) & u_{x}u_{y}\left(1-\text{cos}\phi \right)-u_{z}\text{sin}\phi & u_{x}u_{z}\left(1-\text{cos}\phi \right)+u_{y}\text{sin}\phi \\ u_{y}u_{x}\left(1-\text{cos}\phi \right)+u_{z}\text{sin}\phi & \text{cos}\phi +u_{y}^{2}\left(1-\text{cos}\phi \right) & u_{y}u_{z}\left(1-\text{cos}\phi \right)-u_{x}\text{sin}\phi \\ u_{z}u_{x}\left(1-\text{cos}\phi \right)-u_{y}\text{sin}\phi & u_{z}u_{y}\left(1-\text{cos}\phi \right)+u_{x}\text{sin}\phi & \text{cos}\phi +u_{z}^{2}\left(1-\text{cos}\phi \right) \end{array}\right]$$

The rotation angle $\phi_{j}$ is a function of wavelength, which is expressed as

$$\phi_{j}=\text{GRM}\left[{\left(\frac{\lambda }{1\;m}\right)}^{\alpha }-{\left(\frac{\lambda_{c}}{1\;m}\right)}^{\alpha }\right]\;\text{rad}$$ (6)

where GRM is the “generalized” RM and α is the wavelength-dependent index. $\lambda_{c}$ is the corresponding wavelength at central observation frequency of 784 MHz. We used the MULTINEST sampler to derive the posterior distributions and parameter estimations for fitting the frequency-dependent polarization vector variation. The resulting GRMs for SP1, SP2, and SP3 are $20.01_{-8.8}^{+0.45}$, $4.16_{-0.49}^{+0.44}$, and $45.97_{-0.6}^{+1.0}$, respectively. The corresponding wavelength-dependent indices are $0.35_{-0.24}^{+0.39}$, $0.84_{-0.16}^{+0.23}$, and $0.95_{-0.13}^{+0.17}$. It has been proposed that generalized Faraday rotation could result in phase-resolved variations of RM (*53*). To explore this possibility, we conducted RM fittings for each 2-s data block. Notably, the RMs exhibit significant variations across the pulse phase in P2, particularly during the time interval characterized by peak flux and rapid PA variations (fig. S10). The RM variations should not be attributed to instrument artifacts, as the RM for some pulsars observed with the same instruments remains stable within <1 rad m^−2^ over the timescale of days (*68*). However, the possibility that these variations arise from instantaneous changes in magnetic fields along the line of sight cannot be excluded.

### Down-drifting polarization conversion

In the P2 pulse, a down-drifting linear-to-circular polarization conversion structure was observed in the Stokes spectrum. This down-drifting conversion cannot be attributed to polarization calibration issues, as the system is expected to remain stable at such short timescales. Moreover, it is challenging to produce a drifting feature through calibration issues. To further investigate this behavior, we selected the full-polarization data within the time span of 85 to 90 s in P2. The Faraday rotation was corrected with the RM of 530.67 rad m^−2^. The resulting Stokes spectrum is shown in Fig. 4, where a down-drifting band with linear-to-circular polarization conversion can be observed. To characterize this behavior, we created residual plots of the frequency-dependent PA and CP/LP fraction for successive 0.2-s segments (fig. S11). The PAs in segments exhibit pulsed increases with the central time drifting along the frequency. The CP fractions in segments show drifting pulsed increases, while the LP fractions show drifting pulsed decreases. In addition, the total intensity shows drifting pulsed decreases. To quantify the down-drifting conversion, we fitted the frequency-dependent PA and CP/LP fraction variations using Gaussian shapes, where the central frequency ν of the pulsed variation is modeled as

$$\frac{\nu }{1\;\text{GHz}}={\left[\frac{t-t_{c}}{D/241\;s}+{\left(\frac{\nu_{c}}{1\;\text{GHz}}\right)}^{-\beta }\right]}^{-1/\beta }$$ (7)

where *t* is the central time of a segment, $t_{c}$ is the mean time of the entire time span, and $\nu_{c}$ is the corresponding central frequency of the pulsed variation at $t_{c}$. β is the frequency-dependent relation index of the drifting. The down-drifting relation model is similar to the dispersion relation with = 2, where *D* is the value of the “equivalent” DM. For frequency-dependent PA variations and LP/CP variations, we used different central frequencies $v_{c}$. The resulting *D* is $390_{-55}^{+217}$ with the corresponding $\beta =1.95_{-0.61}^{+0.03}$. To characterize the total intensity decreases, we modeled the total intensity variations using Gaussian templates with the same time-frequency drifting relation obtained in the LP/CP fitting. The average decrease in total intensity is approximately 50%.

## Supplementary Materials

### This PDF file includes:

Supplementary Text Figs. S1 to S11 Table S1 References
