# Supplementary material for: A highly magnetized long-period radio transient exhibiting unusual emission features
Source: Sci Adv. 2025 Jan 17;11(3):eadp6351. doi: 10.1126/sciadv.adp6351 (PMC11740945; doi:10.1126/sciadv.adp6351)
Supplement: Supplementary file 1 — Supplementary Text Figs. S1 to S11 Table S1 References [file sciadv.adp6351_sm.pdf]

Science Advances
AAAS

Supplementary Materials for

A highly magnetized long-period radio transient exhibiting unusual emission features

Yunpeng Men *et al.*

Corresponding author: Natasha Hurley-Walker, nhw@icrar.org; Ewan Barr, ebarr@mpifr-bonn.mpg.de; Yunpeng Men, ypmen@mpifr-bonn.mpg.de

Sci. Adv. **11**, eadp6351 (2025)
DOI: 10.1126/sciadv.adp6351

This PDF file includes:

Supplementary Text
Figs. S1 to S11
Table S1
References

Supplementary Text

Theoretical interpretation

The observations of GPM J1839–10 exhibits a wide range of radio emission properties: (a) Orthogonal polarization modes (OPMs), where smooth position angle (PA) swings are interrupted by approximately 90-degree transitions; (b) Significant circular polarization, sometimes reaching tens of percent; (c) Association of the PA of linear polarization with the sign change of circular polarization; (d) Linear-to-circular polarization conversion; (e) Down-drifting polarization conversion, where the linear polarization converts to circular polarization in a down-drifting frequency band. Among these properties, OPMs, significant circular polarization, and its association with rapid PA changes are common in normal pulsars, millisecond pulsars, and radio magnetars, despite differences in magnetic field structures and magnetosphere sizes (16). These radio emission characteristics can be attributed to either intrinsic emission mechanisms or propagation effects within the magnetosphere, or external influences such as a magneto-ionic environment in a binary system or a supernova remnant. Below, we explore possible interpretations of these radio properties in various scenarios.

Orthogonal polarization modes In the propagation of natural waves through the ultrarelativistic, highly magnetized plasma in a pulsar magnetosphere, only three normal wave modes are allowed (16, 69): the extraordinary X-mode, where the electric field vector is perpendicular to both the wave vector and the magnetic field; the superluminal mode; and the subluminal Alfvén mode, with the electric field in the plane of the wave vector and the magnetic field. The Alfvén mode experiences Landau damping for oblique propagation along magnetic field lines, so the observed orthogonal polarization modes (OPMs) consist of the extraordinary X-mode and the superluminal O-mode. The occurrence of OPMs can be explained by propagation effects in the magnetosphere: (a) Cyclotron absorption or induced scattering occasionally occurs

for one of the modes (70) (b) The two modes are separated in space and angle due to different refractive indices (71); (c) The superluminal O-mode transforms into the extraordinary X-mode (69).

Circular polarization and its association with linear polarization Significant circular polarization is observed in the radio emission of GPM J1839–10. This circular polarization can be produced by intrinsic emission mechanisms within the magnetosphere or by propagation effects inside or outside the magnetosphere (72). The coherent radio emission in the magnetosphere is proposed to arise from coherent curvature radiation by charged bunches with high Lorentz factors or from coherent inverse Compton scattering (ICS). In the curvature radiation scenario, circular polarization can be produced when the line-of-sight is off the curved magnetic field plane, which can also result in an antisymmetric profile of circular polarization associated with a rapid position angle change (44, 72). However, the circular polarized radio emission of GPM J1839–10 is also observed without an antisymmetric profile or associated linear polarization, indicating the need for additional mechanisms to account for the observed circular polarization. The coherent ICS process can occur in the magnetosphere when low-frequency electromagnetic waves produced by inner gap sparking are scattered by charged bunches (73, 74). This scattering can generate circular polarization in an off-beam geometry because the electric fields of the scattered waves can have different phases due to the spatial distribution of electrons within the charged bunches (72). Given that GPM J1839–10 has an ultra-long period, the Goldreich-Julian density is very low, significantly reducing the total number of net charges in one bunch. Therefore, a twisted magnetic field might be required rather than a normal dipole magnetic field, as proposed to revise the death line of long-period radio transients (8).

Circular polarization can also be generated through propagation effects. Within the magnetosphere, cyclotron absorption between the left and right circular polarized radio emissions can differ, producing net circular polarization, which requires the electrons and positrons to have an

asymmetric energy distribution. Outside the magnetosphere, it has been noted that synchrotron maser and absorption processes struggle to generate a high degree of circular polarization (72). Cyclotron absorption, on the other hand, is an efficient mechanism for producing high degrees of circular polarization in dense magneto-ionic environments (72). For cyclotron absorption to occur, the magnetic field strength in the magneto-ionic region should be $B \approx \gamma(1-\beta\cos\theta)\frac{2\pi\nu m_e c}{e}$, where θ is the angle between the wave's incident direction and the electron's motion direction. Assuming the radio waves propagate parallel to the magnetic field, $\theta \approx 0°$, the magnetic field strength $B \approx \frac{180\,\mathrm{G}}{\gamma}\frac{\nu}{1\,\mathrm{GHz}}$. For non-parallel incident waves, such as $\theta = 30°$, the magnetic field strength could be underestimated, yielding $B = 48\,\gamma\,\mathrm{G}$, corresponding to a smaller magnetosphere radius. Optical observations suggest the possible presence of a main sequence star with a spectral type ranging from mid-K to mid-M (2), which disfavors highly magnetized companions such as Be-stars or O-stars (72). Therefore, relativistic plasma in the magneto-ionic environment is required for cyclotron absorption in such a binary system. However, This condition can be satisfied within the magnetosphere of a neutron star, where the Lorentz factor $\gamma > 100$, and the magnetic field strength is $B \approx 4 \times 10^{-7}\left(\frac{B_{\mathrm{surf}}}{10^{14}\,\mathrm{G}}\right)\left(\frac{r}{r_{\mathrm{c}}}\right)^{-3}$ G, with the light-cylinder radius of GPM J1839–10 is $r_{\mathrm{c}} = \frac{Pc}{2\pi} \approx 6.3 \times 10^{7}\,\mathrm{km}$. Under these constraints, the propagation effect emerged at a radius of $< 0.005\,r_{\mathrm{c}}$, resulting in a small pulse duty cycle in a dipole magnetic field, which is inconsistent with observations. This indicates that the magnetic field configuration of GPM J1839–10 might be more complex. Another mechanism for generating circular polarization is the Faraday conversion effect. Faraday conversion within the magnetosphere is suggested to produce symmetric profiles of circular polarization (44), which may account for some of the circular polarized radio emissions observed in GPM J1839–10.

Linear-to-circular polarization conversion Linear-to-circular polarization conversion occurs when radio waves propagating through a birefringent medium, manifesting as elliptically or linearly polarized natural wave modes (55,75), which is called Faraday conversion. The exis-

tence of magnetic field perpendicular to the wave direction can result in elliptically natural wave modes (55, 75). The wavelength-dependent indices can vary under different plasma conditions, for instance, $\alpha = 3$ in the relativistic pair-plasma (75), and $\alpha = 1-2$ in the near-wind of a magnetar (76). In the scenario of radio waves propagating through a birefringent magneto-ionetic medium, Faraday conversion becomes significant when the Larmor frequency ν_B is comparable to the radio frequency ν/γ, where the required magnetic field strength is $B \approx \frac{360\,\mathrm{G}}{\gamma}\frac{\nu}{1\,\mathrm{GHz}}$ (50, 55, 77). This provides a rough estimate of the magnetic field strength in the region where the conversions take place, $\gamma B \sim 300\,\mathrm{G}$, which aligns with the estimate from cyclotron absorption. However, it is important to note that cyclotron absorption occurs when radio waves propagate parallel to the magnetic field, while Faraday conversion occurs when they propagate perpendicular to the magnetic field. These propagation effects are therefore expected to occur in distinct regions.

Linear-to-circular polarization conversion can arise from the radio wave propagation effects in the magnetosphere (78), which can also explain its phased-related variation observed in GPM J1839–10. It has been suggested that linear-to-circular polarization conversion can occur in the near-wind of a magnetar with a wavelength-dependent index of 1-2 (76), which can be applied to the observed behavior of GPM J1839–10. As the calculations in the previous section, the conditions for a magneto-ionic medium can be satisfied within the magnetosphere. Polarization conversion can also occur when radio emission passes through the solar wind of a highly magnetized companion, such as a Be-star or a supernova remnant (72). However, optical observations disfavor the scenario of a highly magnetized companion (2). In both cases, the magnetic field strength should not exceed 100 G, implying that the presence of relativistic plasma is required for the observed effects.

The linear-to-circular polarization conversion could also be generated from the multi-path scattering effects when radio waves pass through a magnetized screen (79). The required

condition of RM induced by the screen, $\mathrm{RM_s} \gtrsim 10\left(\frac{\nu}{1\,\mathrm{GHz}}\right)^2\,\mathrm{rad\,m^{-2}}$, can be satisfied for GPM J1839–10. The variability timescale can be estimated as, $t_{\rm scr,var} > (6.7\,\mathrm{s})\nu^{1/2}_{\rm co,100Hz}\nu^{-1}_{\rm 1GHz}d^{1/2}_{\rm 5.7kpc}v^{-1}_{\rm max,7}$, where the correlation bandwidth $\nu_{\rm co}$ should exceed 100 Hz, given the shortest pulse width is less than 10 ms, because scattering tails are not observed in the short-time structures. This scenario is difficult to explain the various frequency-dependent relationships in the polarization conversion in short time scales. The timescale associated with the magnetized screen makes it difficult to account for the rapid PA changes occurring within hundreds of milliseconds. However, this scenario remains applicable during phases with circularly polarized emission and nearly constant PAs.

Down-drifting polarization conversion The spectra of GPM J1839–10 exhibited an unusual emission feature of down-drifting polarization conversion. Within a drifting sub-band, the emission converted from linear polarization to circular polarization, while outside this sub-band, no conversion occurred. Additionally, within the sub-band, the total intensity decreased by an average of about 50%. This could be evidence of cyclotron absorption, where left and right circular polarization were asymmetrically absorbed. The drifting in the absorption frequencies could be attributed to different magnetic field strengths in the absorption region. In this scenario, the magnetic field strength can be estimated using the cyclotron frequency $B = \frac{360\,\mathrm{G}}{\gamma}\frac{\nu}{1\,\mathrm{GHz}}$ (72).

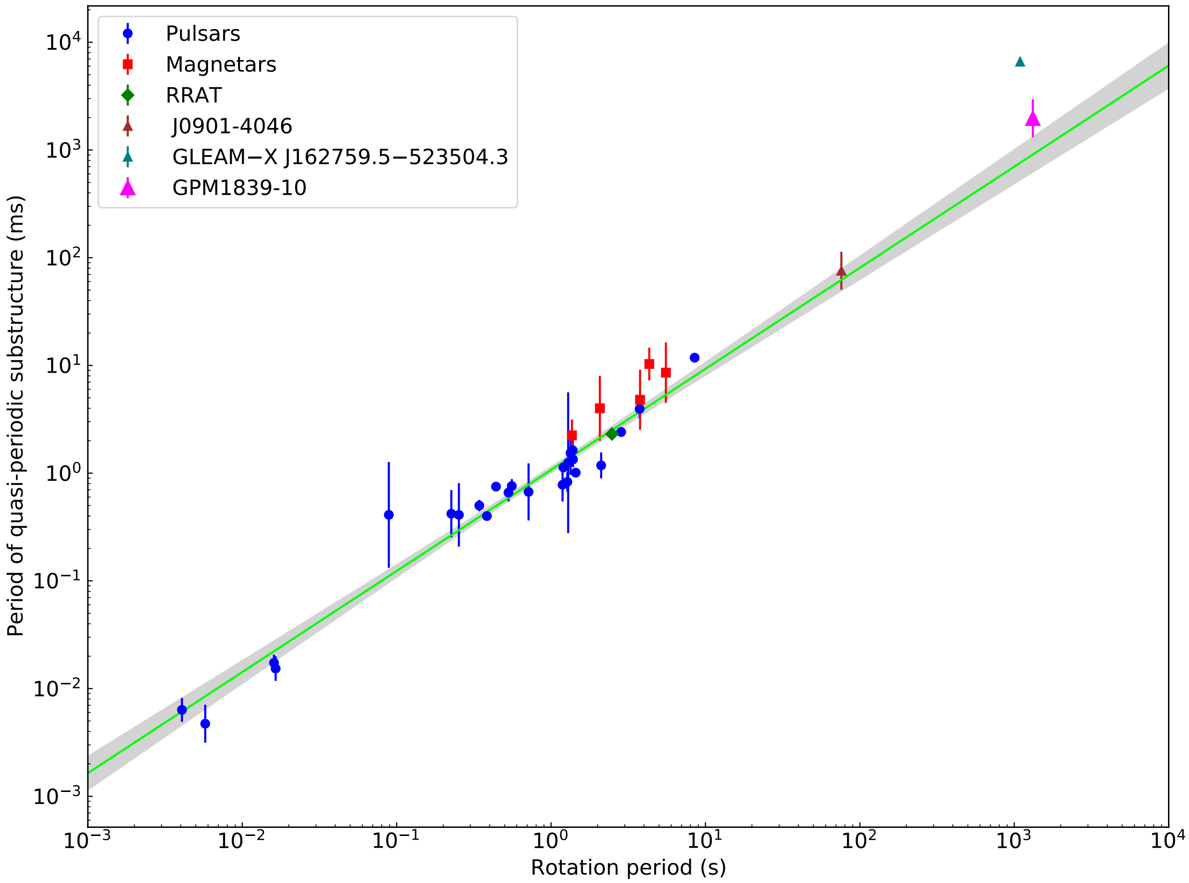

Figure S1: **Relationship between quasi-periodicity and the observed sub-structure as a function of the neutron star rotation period.** It was delineated in the reference by (26). The parameters of GPM J1839–10 observed in the P2 pulse are denoted by the pink upper triangle. We re-estimated the 1-sigma confidence interval using the maximum likelihood estimation method.

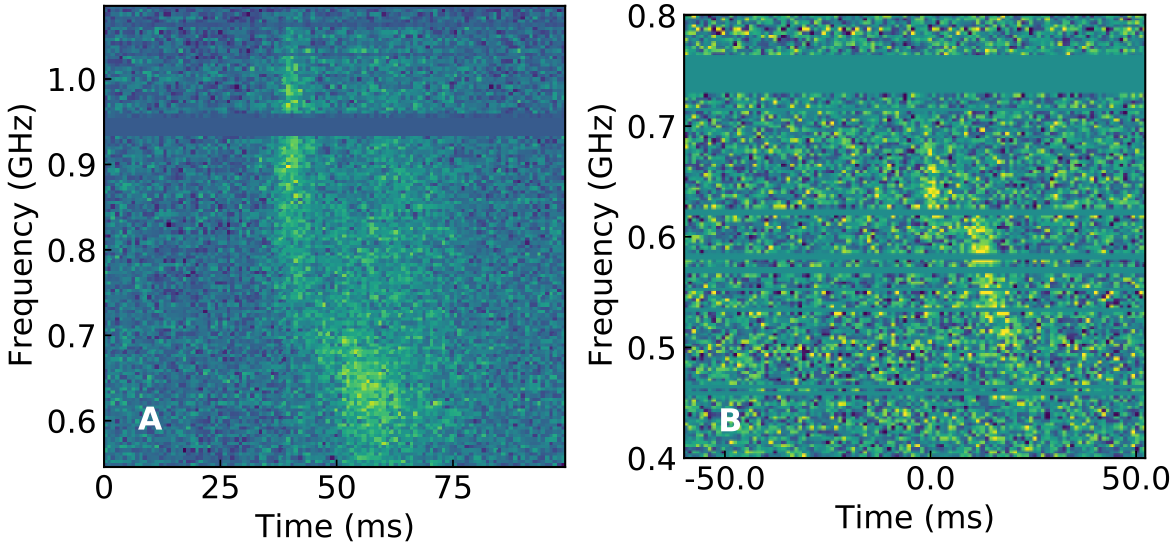

Figure S2: **Comparison of spectra of the down-drifting structures between GPM J1839–10 and FRB 20201229C**. (A) Spectrum of the down-drifting sub-structure within the P3 pulse of GPM J1839–10. (B) Spectrum of the repeating source FRB 20201229C (80).

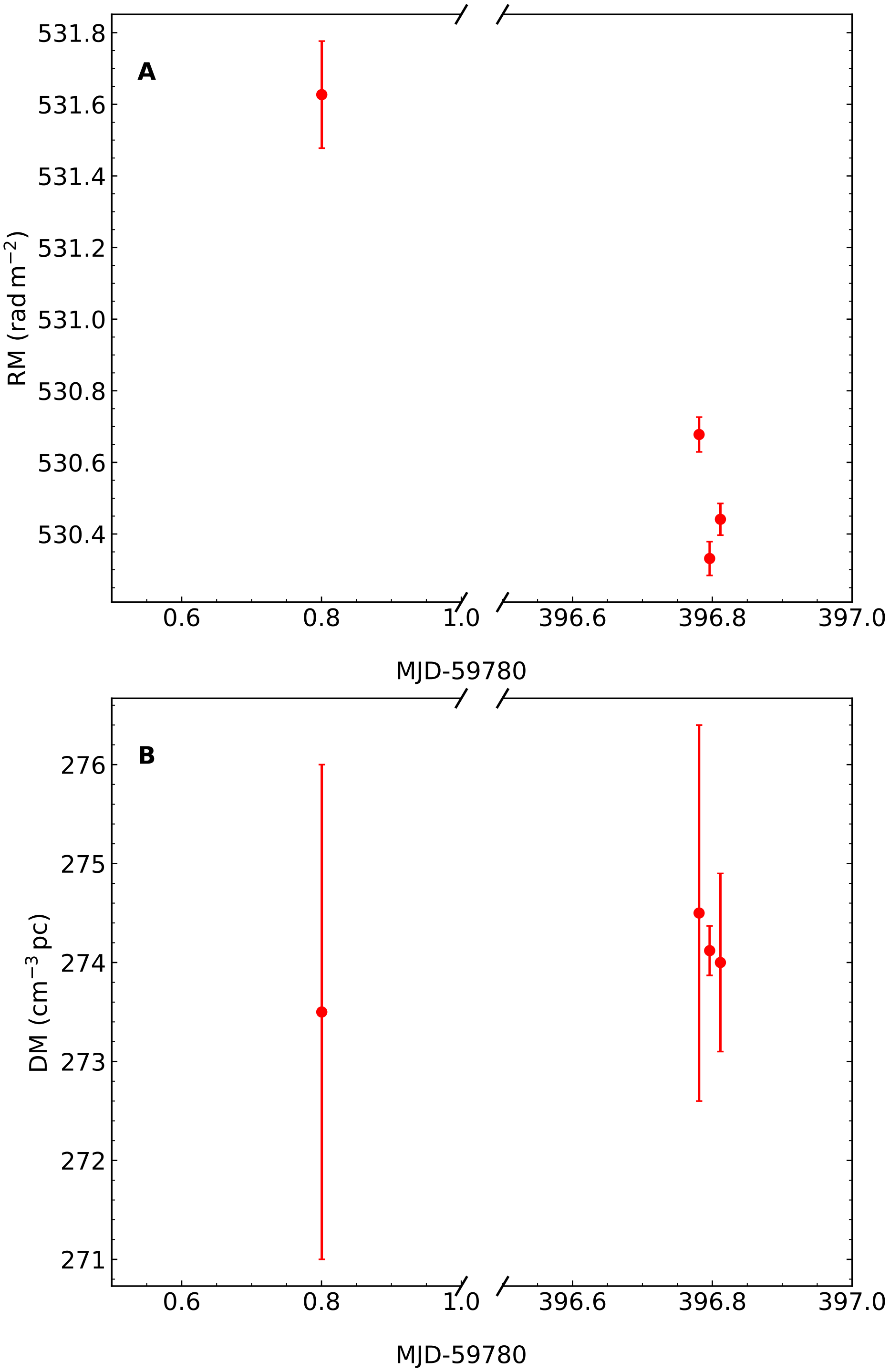

Figure S3: **Variations in RMs and DMs of GPM J1839–10 observed on July 20, 2022 (UT) and August 20, 2023 (UT).** (A) RM variations with correction for the ionosphere's RM contribution (see materials and methods). (B) DM variations.

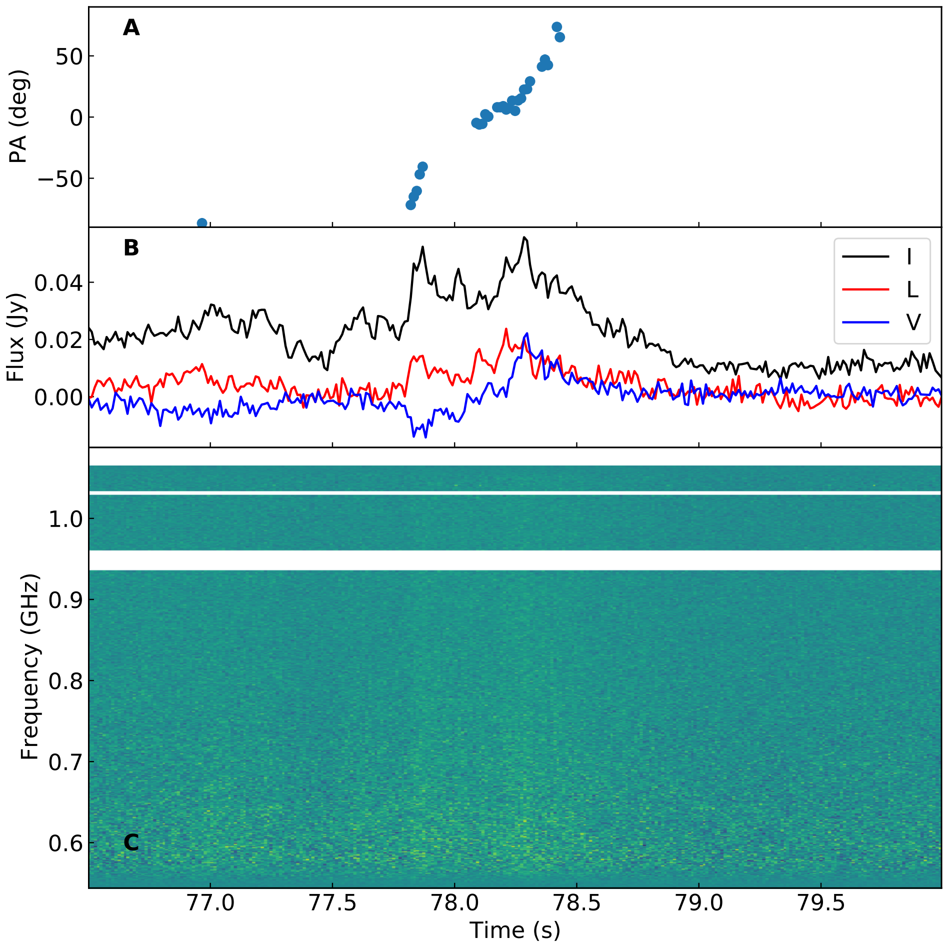

Figure S4: **Dynamic spectra of a sub-pulse exhibiting sign change in circular polarization coinciding with rapid PA change.** The sub-pulse is in period P3. (A) PA curve. (B) Total intensity (black), linear polarization intensity (red) and circular polarization intensity (blue). (C) Dynamic spectrum.

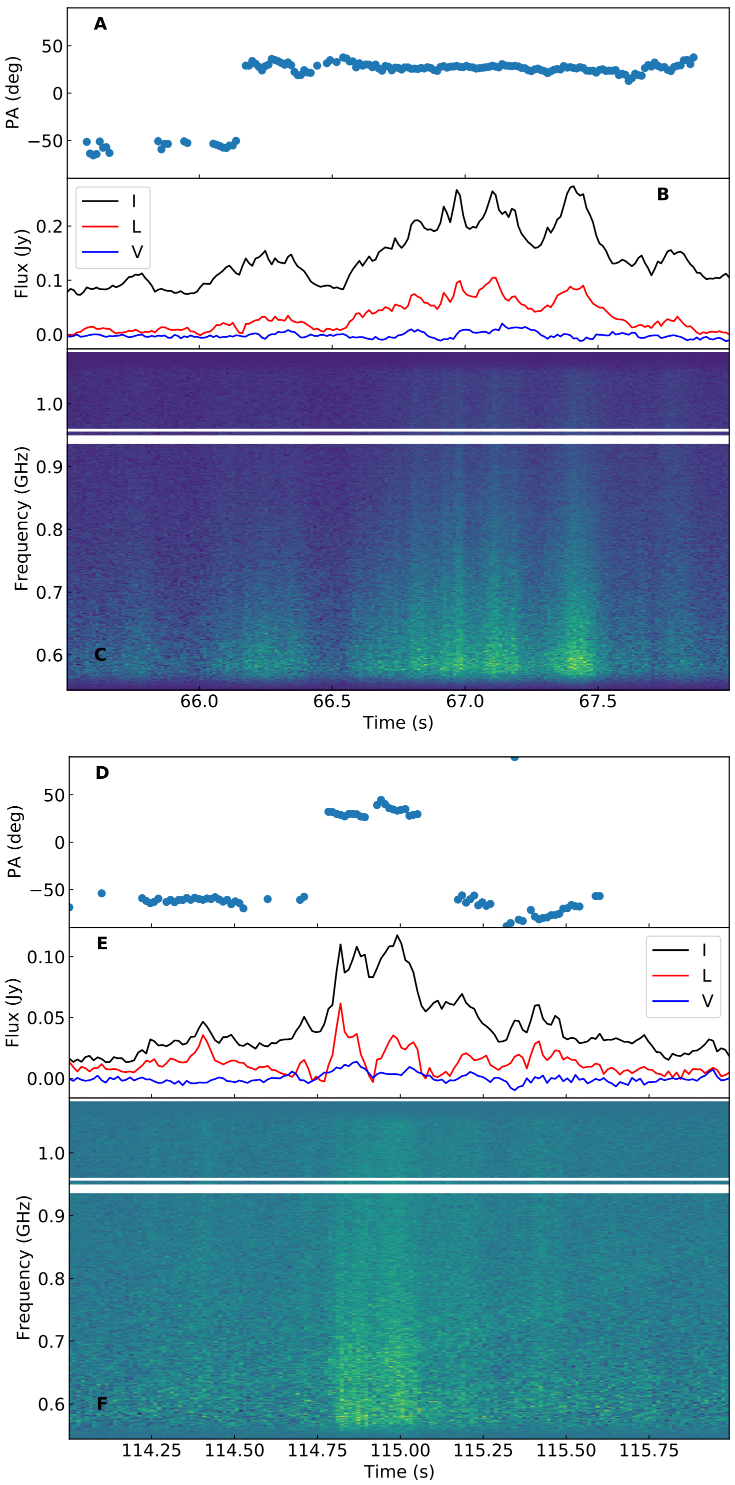

Figure S5: **Dynamic spectra of sub-pulses with orthogonal jumps.** Both sub-pulses are in period P2. (A) (D) PA curve. (B) (E) Total intensity (black), linear polarization intensity (red) and circular polarization intensity (blue). (C) (F) Dynamic spectra.

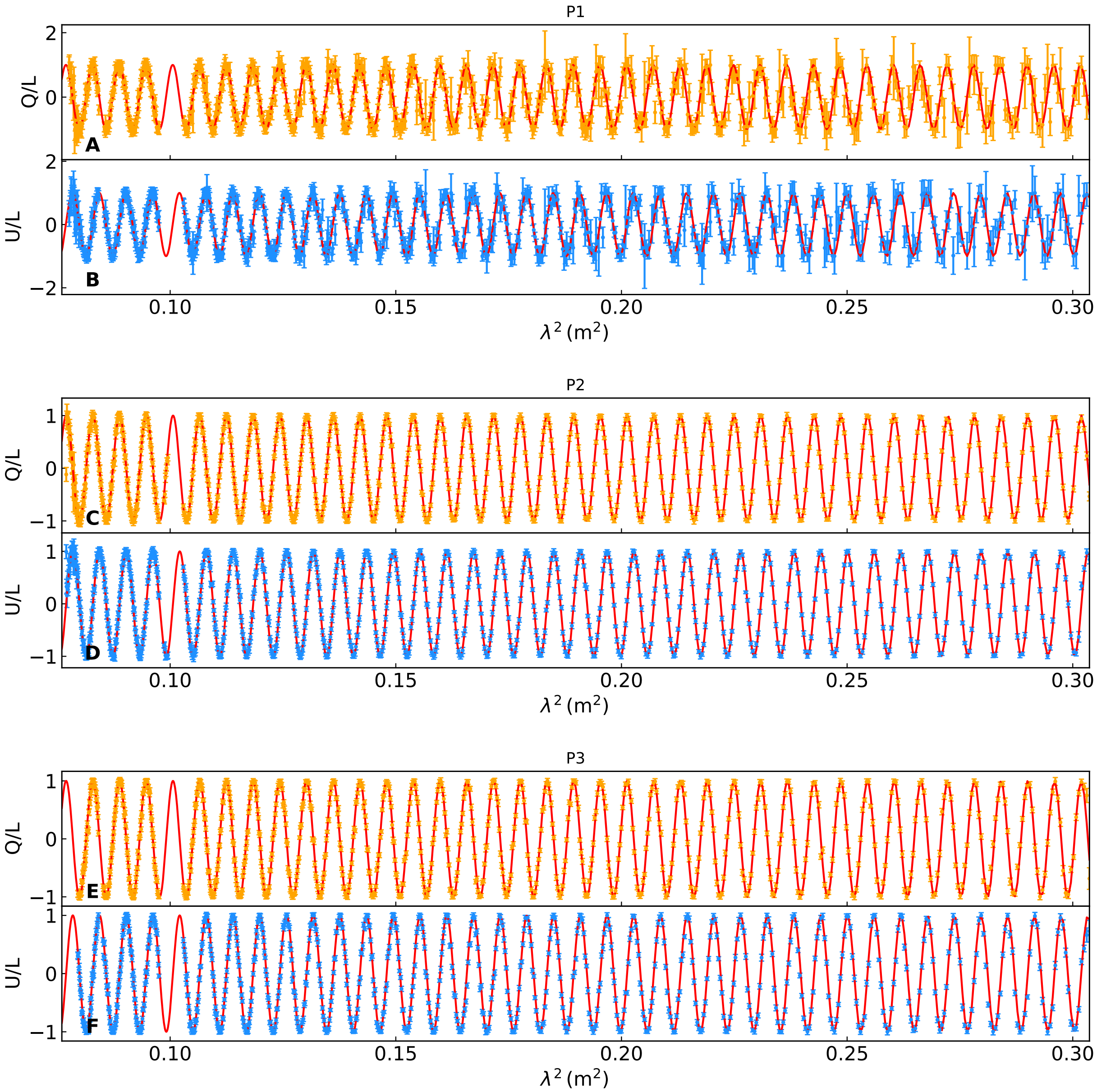

Figure S6: **Fitted curves for Stokes Q and U as a function of the square of the wavelength derived from fitting the QU spectrum.** (A) (C) (E) Fitting for the normalized Stokes Q intensity. (B) (D) (F) Fitting for the normalized Stokes U intensity.

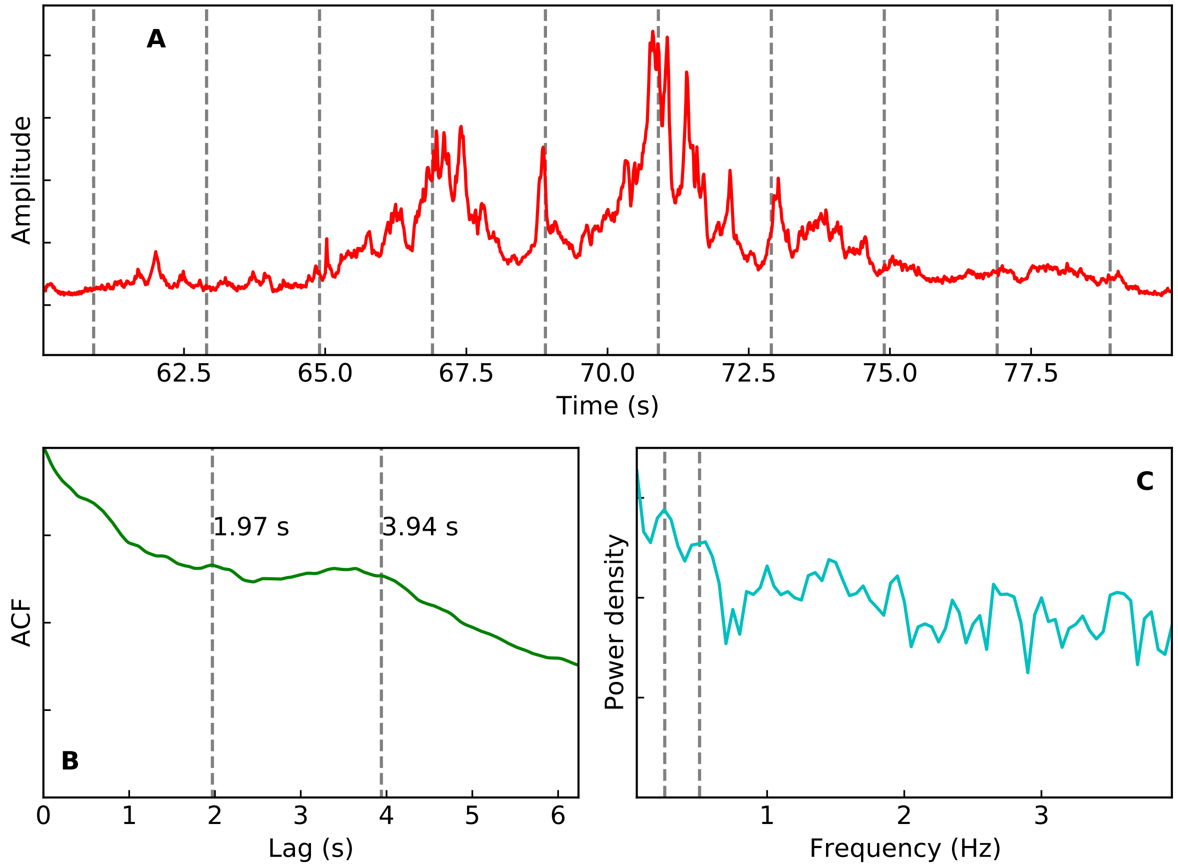

Figure S7: **Periodicity analysis of the P2 pulse.** (A) Total intensity profile of the P2 pulse within the time range of 60-80 s. (B) Auto-correlation function (ACF). The gray dashed lines highlight the peak around 1.97 s and its double period, 3.94 s. (C) Power spectral density (PSD). The corresponding frequencies at 1.97 s and 3.94 s are marked by the gray dashed lines.

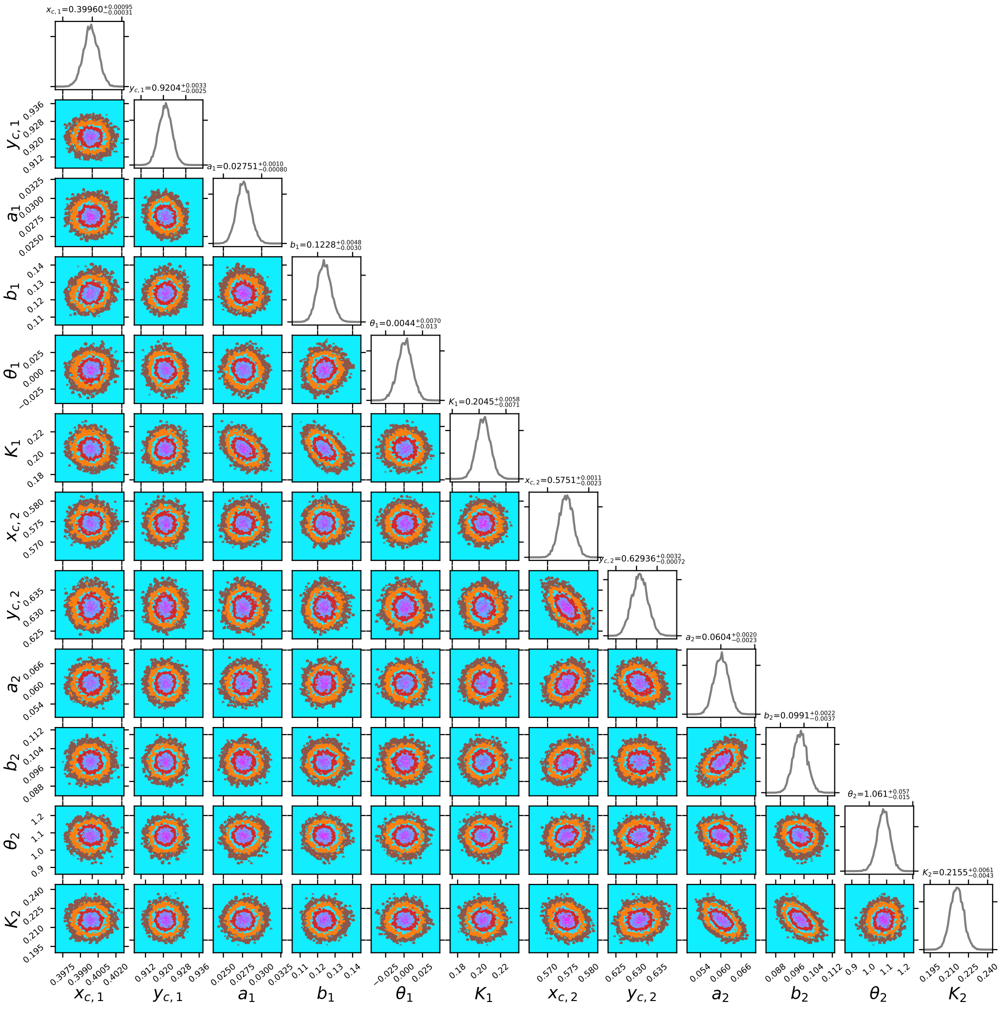

Figure S8: **Posterior distributions illustrating the parameter estimation from spectrum fitting of the down-drifting sub-structure.**

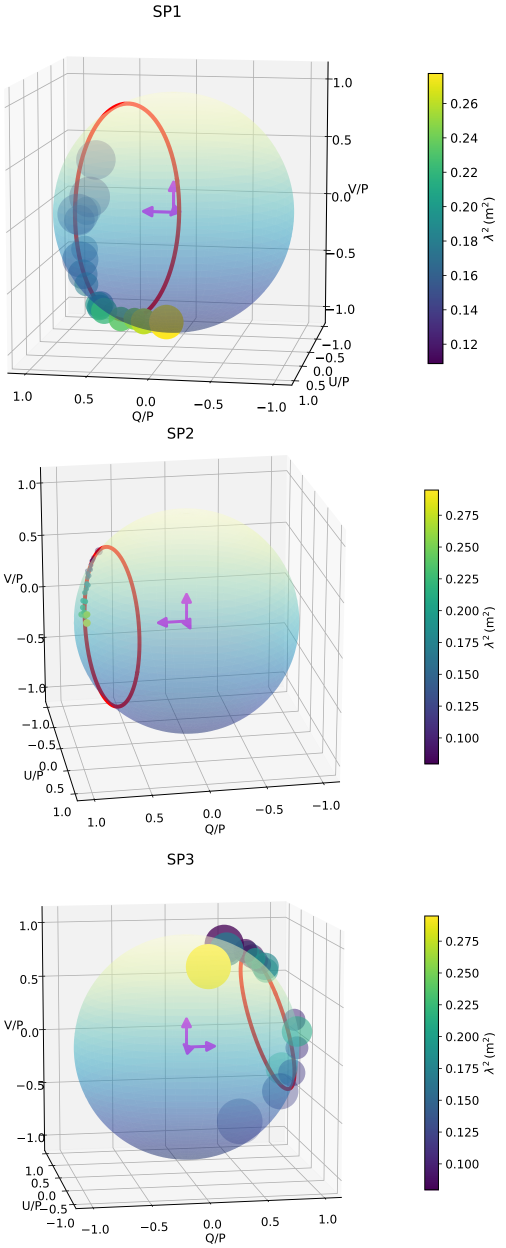

Figure S9: **The Poincaré sphere representation of the polarization vectors for the three sub-pulses, displaying linear-to-circular polarization conversion.** The red circle denotes the Faraday conversion fitting (see materials and methods). The size of each point corresponds to the error bars of the polarization vectors on the Poincaré sphere.

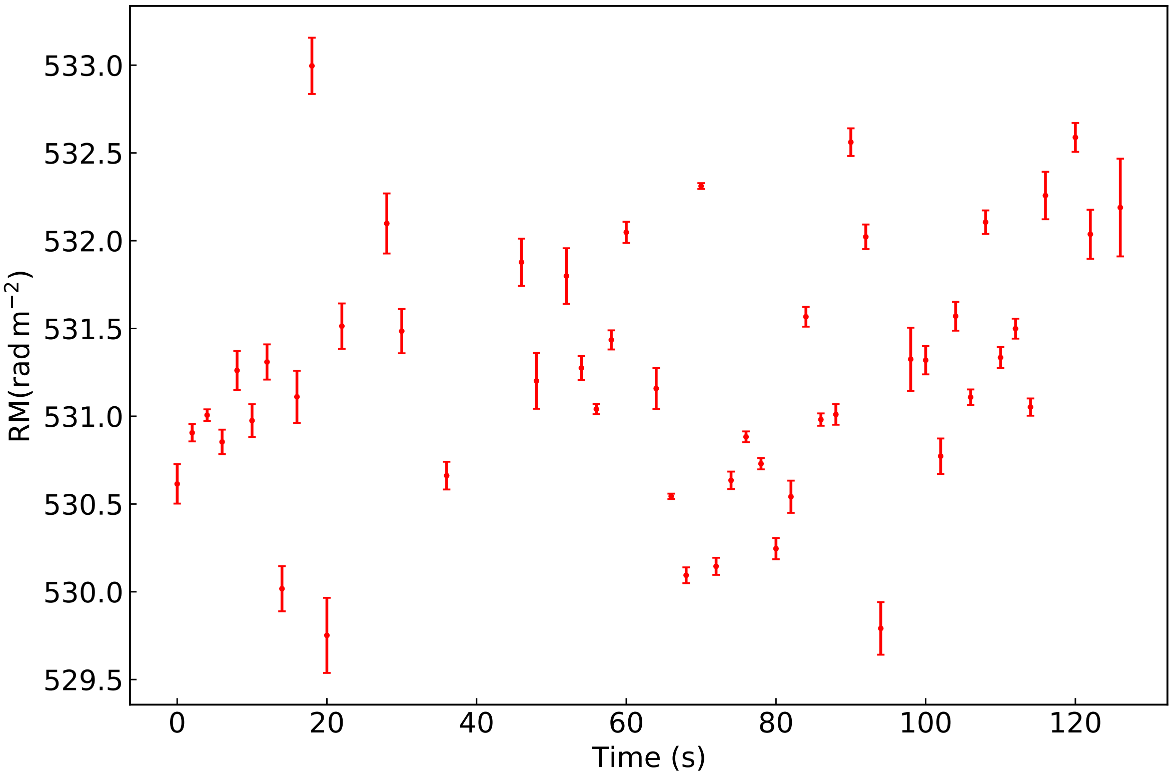

Figure S10: **Time-resolved RM variation.** The RM measurement was conducted for each two-second data block. Data points displaying an RM deviation exceeding $2\,\mathrm{rad\,m^{-2}}$ from the baseline of $531\,\mathrm{rad\,m^{-2}}$ were removed due to the weak linear polarized intensity.

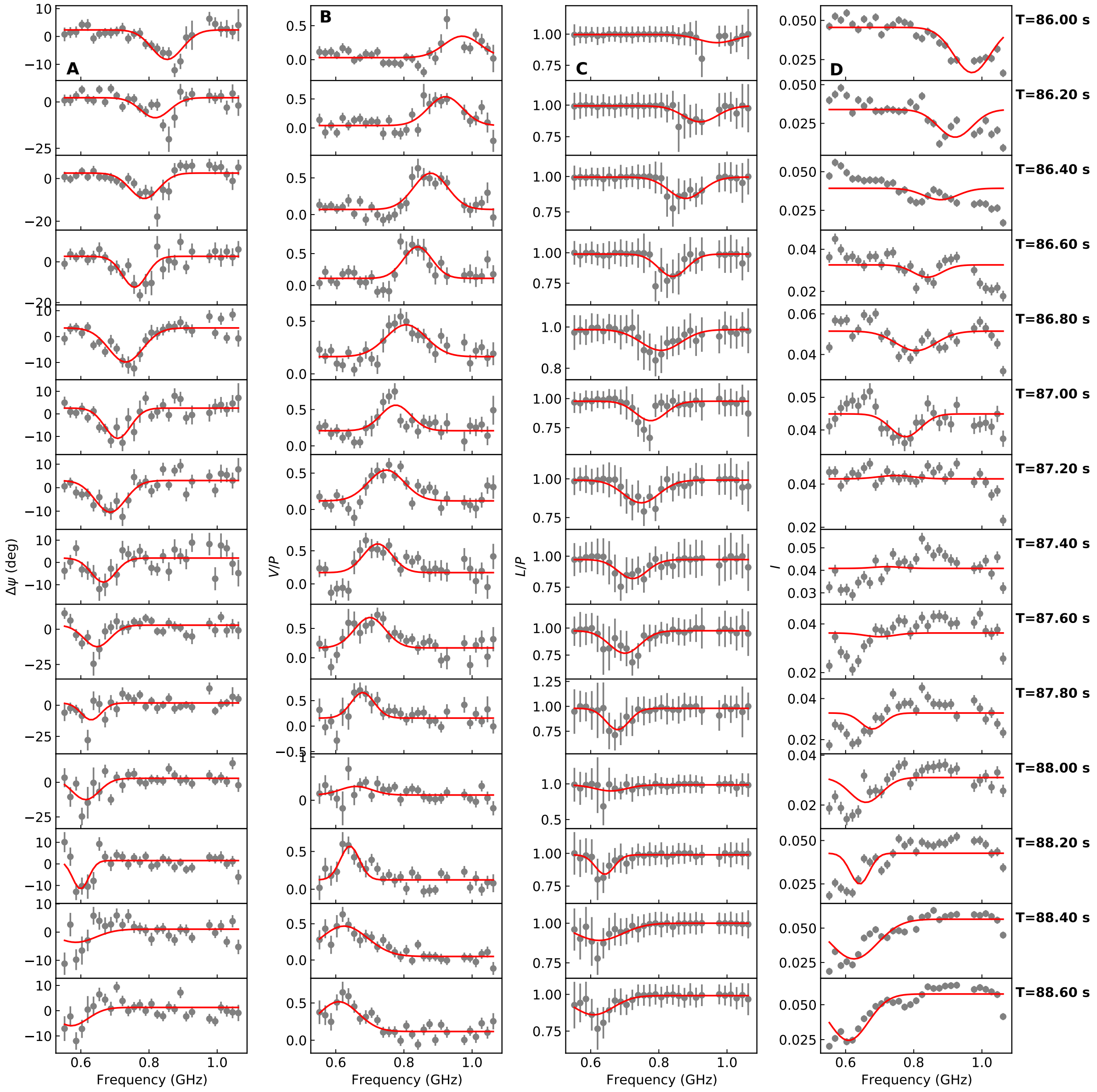

Figure S11: **Frequency-dependent PA and CP/LP variations for successive 0.2-second data segments.** (A) Fitting of PA variation. (B) Fitting of frequency-dependent circular polarization variation. (C) Fitting of frequency-dependent linear polarization variation. (D) Fitting of frequency-dependent total intensity variation. The red solid lines represent the fitted Gaussian shapes with a frequency-dependent relation (see materials and methods).

Table S1: **Follow-up observation schedule of GPM J1839–10.** The columns provide the start time of the session, the central observing frequency f, bandwidth Δf, integration time $t_{\rm obs}$ and time resolution δt of the recorded data. Sessions with detected pulsed radio emission are indicated by an asterisk.

Session	Start Time	f (MHz)	Δf (MHz)	$t_{\rm obs}$ (min)	δt (μs)
1	2023-08-20 14:17:02	816	544	10	15
2	2023-08-20 14:39:02	816	544	10	15
3	2023-08-20 15:01:03	816	544	10	15
4	2023-08-20 15:23:03	816	544	10	15
5	2023-08-20 15:45:05	816	544	10	15
6	2023-08-20 16:07:06	816	544	10	15
7	2023-08-20 16:29:07	816	544	10	15
8	2023-08-20 16:56:03	816	544	10	15
9	2023-08-20 17:15:31	816	544	10	15
10	2023-08-20 17:37:36	816	544	10	15
11	2023-08-20 17:59:43	816	544	10	15
12	2023-08-20 18:21:53	816	544	10	15
13*	2023-08-20 18:44:02	816	544	10	15
14*	2023-08-20 19:06:12	816	544	10	15
15*	2023-08-20 19:28:22	816	544	10	15

REFERENCES AND NOTES

1. N. Hurley-Walker, X. Zhang, A. Bahramian, S. J. McSweeney, T. N. O'Doherty, P. J. Hancock, J. S. Morgan, G. E. Anderson, G. H. Heald, T. J. Galvin, A radio transient with unusually slow periodic emission. *Nature* **601**, 526–530 (2022).

2. N. Hurley-Walker, N. Rea, S. J. McSweeney, B. W. Meyers, E. Lenc, I. Heywood, S. D. Hyman, Y. P. Men, T. E. Clarke, F. Coti Zelati, D. C. Price, C. Horváth, T. J. Galvin, G. E. Anderson, A. Bahramian, E. D. Barr, N. D. R. Bhat, M. Caleb, M. Dall'Ora, D. de Martino, S. Giacintucci, J. S. Morgan, K. M. Rajwade, B. Stappers, A. Williams, A long-period radio transient active for three decades. *Nature* **619**, 487–490 (2023).

3. M. Caleb, E. Lenc, D. L. Kaplan, T. Murphy, Y. P. Men, R. M. Shannon, L. Ferrario, K. M. Rajwade, T. E. Clarke, S. Giacintucci, N. Hurley-Walker, S. D. Hyman, M. E. Lower, S. McSweeney, V. Ravi, E. D. Barr, S. Buchner, C. M. L. Flynn, J. W. T. Hessels, M. Kramer, J. Pritchard, B. W. Stappers, An emission-state-switching radio transient with a 54-minute period. *Nat. Astron.* **8**, 1159–1168 (2024).

4. P. Beniamini, Z. Wadiasingh, J. Hare, K. M. Rajwade, G. Younes, A. J. van der Horst, Evidence for an abundant old population of Galactic ultra-long period magnetars and implications for fast radio bursts. *Mon. Not. R. Astron. Soc.* **520**, 1872–1894 (2023).

5. Y. Qu, B. Zhang, Magnetic interaction in white dwarf binaries as mechanism for long-period radio transients. arXiv:2409.05978 [astro-ph.HE] (2024).

6. N. Rea, N. Hurley-Walker, C. Pardo-Araujo, M. Ronchi, V. Graber, F. Coti Zelati, D. de Martino, A. Bahramian, S. J. McSweeney, T. J. Galvin, S. D. Hyman, M. Dall'Ora, Long-period radio pulsars: Population study in the neutron star and white dwarf rotating dipole scenarios. *Astrophys. J.* **961**, 214 (2024).

7. A. Loeb, D. Maoz, A hot subdwarf model for the 18.18 minutes Pulsar GLEAM-X. *Res. Notes AAS* **6**, 27 (2022).

8. H. Tong, On the nature of long period radio pulsar GPM J1839-10: Death line and pulse width. *Res. Astron. Astrophys.* **23**, 125018 (2023).

9. V. M. Kaspi, A. M. Beloborodov, Magnetars. *Annu. Rev. Astron. Astrophys* **55**, 261–301 (2017).

10. F. Camilo, S. M. Ransom, J. P. Halpern, J. Reynolds, D. J. Helfand, N. Zimmerman, J. Sarkissian, Transient pulsed radio emission from a magnetar. *Nature* **442**, 892–895 (2006).

11. F. Camilo, S. M. Ransom, J. P. Halpern, J. Reynolds, 1E 1547.0-5408: A radio-emitting magnetar with a rotation period of 2 seconds. *Astrophys. J.* **666**, L93–L96 (2007).

12. L. Levin, M. Bailes, S. Bates, N. D. R. Bhat, M. Burgay, S. Burke-Spolaor, N. D'Amico, S. Johnston, M. Keith, M. Kramer, S. Milia, A. Possenti, N. Rea, B. Stappers, W. van Straten, A radio-loud magnetar in x-ray quiescence. *Astrophys. J.* **721**, L33–L37 (2010).

13. R. P. Eatough, H. Falcke, R. Karuppusamy, K. J. Lee, D. J. Champion, E. F. Keane, G. Desvignes, D. H. F. M. Schnitzeler, L. G. Spitler, M. Kramer, B. Klein, C. Bassa, G. C. Bower, A. Brunthaler, I. Cognard, A. T. Deller, P. B. Demorest, P. C. C. Freire, A. Kraus, A. G. Lyne, A. Noutsos, B. Stappers, N. Wex, A strong magnetic field around the supermassive black hole at the centre of the Galaxy. *Nature* **501**, 391–394 (2013).

14. D. Champion, I. Cognard, M. Cruces, G. Desvignes, F. Jankowski, R. Karuppusamy, M. J. Keith, C. Kouveliotou, M. Kramer, K. Liu, A. G. Lyne, M. B. Mickaliger, B. O'Connor, A. Parthasarathy, N. Porayko, K. Rajwade, B. W. Stappers, P. Torne, A. J. van der Horst, P. Weltevrede, High-cadence observations and variable spin behaviour of magnetar Swift J1818.0-1607 after its outburst. *Mon. Not. R. Astron. Soc.* **498**, 6044–6056 (2020).

15. CHIME/FRB Collaboration, B. C. Andersen, K. M. Bandura, M. Bhardwaj, A. Bij, M. M. Boyce, P. J. Boyle, C. Brar, T. Cassanelli, P. Chawla, T. Chen, J. F. Cliche, A. Cook, D. Cubranic, A. P. Curtin, N. T. Denman, M. Dobbs, F. Q. Dong, M. Fandino, E. Fonseca, B. M. Gaensler, U. Giri, D. C. Good, M. Halpern, A. S. Hill, G. F. Hinshaw, C. Höfer, A. Josephy, J. W. Kania, V. M. Kaspi, T. L. Landecker, C. Leung, D. Z. Li, H. H. Lin, K. W. Masui, R. McKinven, J. Mena-Parra, M. Merryfield, B. W. Meyers, D. Michilli, N. Milutinovic, A. Mirhosseini, M. Münchmeyer, A. Naidu, L. B. Newburgh, C. Ng, C. Patel, U. L. Pen, T. Pinsonneault-Marotte, Z. Pleunis, B. M. Quine, M. Rafiei-Ravandi, M. Rahman, S. M. Ransom, A. Renard, P. Sanghavi, P. Scholz, J. R. Shaw, K. Shin, S. R. Siegel, S. Singh, R. J.

Smegal, K. M. Smith, I. H. Stairs, C. M. Tan, S. P. Tendulkar, I. Tretyakov, K. Vanderlinde, H. Wang, D. Wulf, A. V. Zwaniga, A bright millisecond-duration radio burst from a Galactic magnetar. *Nature* **587**, 54–58 (2020).

16. A. Philippov, M. Kramer, Pulsar magnetospheres and their radiation. *Annual Rev. in Astron. Astrophys* **60**, 495–558 (2022).

17. D. R. Lorimer, M. Bailes, M. A. McLaughlin, D. J. Narkevic, F. Crawford, A bright millisecond radio burst of extragalactic origin. *Science* **318**, 777 (2007).

18. CHIME/FRB Collaboration, M. Amiri, B. C. Andersen, K. Bandura, S. Berger, M. Bhardwaj, M. M. Boyce, P. J. Boyle, C. Brar, D. Breitman, T. Cassanelli, P. Chawla, T. Chen, J. F. Cliche, A. Cook, D. Cubranic, A. P. Curtin, M. Deng, M. Dobbs, F. A. Dong, G. Eadie, M. Fandino, E. Fonseca, B. M. Gaensler, U. Giri, D. C. Good, M. Halpern, A. S. Hill, G. Hinshaw, A. Josephy, J. F. Kaczmarek, Z. Kader, J. W. Kania, V. M. Kaspi, T. L. Landecker, D. Lang, C. Leung, D. Li, H.-H. Lin, K. W. Masui, R. McKinven, J. Mena-Parra, M. Merryfield, B. W. Meyers, D. Michilli, N. Milutinovic, A. Mirhosseini, M. Münchmeyer, A. Naidu, L. Newburgh, C. Ng, C. Patel, U.-L. Pen, E. Petroff, T. Pinsonneault-Marotte, Z. Pleunis, M. Rafiei-Ravandi, M. Rahman, S. M. Ransom, A. Renard, P. Sanghavi, P. Scholz, J. R. Shaw, K. Shin, S. R. Siegel, A. E. Sikora, S. Singh, K. M. Smith, I. Stairs, C. M. Tan, S. P. Tendulkar, K. Vanderlinde, H. Wang, D. Wulf, A. V. Zwaniga, The first CHIME/FRB fast radio burst catalog. *Astrophys. J. Suppl. Ser.* **257**, 59 (2021).

19. M. P. Snelders, K. Nimmo, J. W. T. Hessels, Z. Bensellam, L. P. Zwaan, P. Chawla, O. S. Ould-Boukattine, F. Kirsten, J. T. Faber, V. Gajjar, Detection of ultra-fast radio bursts from FRB 20121102A. *Nat. Astron.* **7**, 1486–1496 (2023).

20. C. D. Bochenek, V. Ravi, K. V. Belov, G. Hallinan, J. Kocz, S. R. Kulkarni, D. L. McKenna, A fast radio burst associated with a Galactic magnetar. *Nature* **587**, 59–62 (2020).

21. P. Beniamini, Z. Wadiasingh, B. D. Metzger, Periodicity in recurrent fast radio bursts and the origin of ultralong period magnetars. *Mon. Not. R. Astron. Soc.* **496**, 3390–3401 (2020).

22. B. Zhang, A. K. Harding, A. G. Muslimov, Radio pulsar death line revisited: Is PSR J2144-3933 anomalous? *Astrophys. J.* **531**, L135–L138 (2000).

23. J. M. Cordes, Pulsar microstructure: Periodicities, polarization and probes of pulsar magnetospheres. *Aust. J. Phys.* **32**, 9–24 (1979).

24. D. Mitra, M. Arjunwadkar, J. M. Rankin, Polarized quasiperiodic structures in pulsar radio emission reflect temporal modulations of non-stationary plasma flow. *Astrophys. J.* **806**, 236 (2015).

25. J. L. Chen, Z. G. Wen, J. P. Yuan, N. Wang, D. Li, H. G. Wang, W. M. Yan, R. Yuen, P. Wang, Z. Wang, W. W. Zhu, J. R. Niu, C. C. Miao, M. Y. Xue, B. P. Gong, The discovery of a rotating radio transient J1918-0449 with intriguing emission properties with the five-hundred-meter aperture spherical radio telescope. *Astrophys. J.* **934**, 24 (2022).

26. M. Kramer, K. Liu, G. Desvignes, R. Karuppusamy, B. W. Stappers, Quasi-periodic sub-pulse structure as a unifying feature for radio-emitting neutron stars. *Nat. Astron.* **8**, 230–240 (2023).

27. CHIME/FRB Collaboration, A second source of repeating fast radio bursts. *Nature* **566**, 235–238 (2019).

28. CHIME/FRB Collaboration, B. C. Andersen, K. Bandura, M. Bhardwaj, P. Boubel, M. M. Boyce, P. J. Boyle, C. Brar, T. Cassanelli, P. Chawla, D. Cubranic, M. Deng, M. Dobbs, M. Fandino, E. Fonseca, B. M. Gaensler, A. J. Gilbert, U. Giri, D. C. Good, M. Halpern, A. S. Hill, G. Hinshaw, C. Höfer, A. Josephy, V. M. Kaspi, R. Kothes, T. L. Landecker, D. A. Lang, D. Z. Li, H. H. Lin, K. W. Masui, J. Mena-Parra, M. Merryfield, R. Mckinven, D. Michilli, N. Milutinovic, A. Naidu, L. B. Newburgh, C. Ng, C. Patel, U. Pen, T. Pinsonneault-Marotte, Z. Pleunis, M. Rafiei-Ravandi, M. Rahman, S. M. Ransom, A. Renard, P. Scholz, S. R. Siegel, S. Singh, K. M. Smith, I. H. Stairs, S. P. Tendulkar, I. Tretyakov, K. Vanderlinde, P. Yadav, A. V. Zwaniga, CHIME/FRB discovery of eight new repeating fast radio burst sources. *Astrophys. J.* **885**, L24 (2019).

29. J. W. T. Hessels, L. G. Spitler, A. D. Seymour, J. M. Cordes, D. Michilli, R. S. Lynch, K. Gourdji, A. M. Archibald, C. G. Bassa, G. C. Bower, S. Chatterjee, L. Connor, F. Crawford, J. S. Deneva, V. Gajjar, V. M. Kaspi, A. Keimpema, C. J. Law, B. Marcote, M. A. McLaughlin, Z. Paragi, E. Petroff, S. M. Ransom, P. Scholz, B. W. Stappers, S. P. Tendulkar, FRB 121102 bursts show complex time-frequency structure. *Astrophys. J.* **876**, L23 (2019).

30. C. K. Day, A. T. Deller, R. M. Shannon, H. Qiu, K. W. Bannister, S. Bhandari, R. Ekers, C. Flynn, C. W. James, J.-P. Macquart, E. K. Mahony, C. J. Phillips, J. Xavier Prochaska, High time resolution and polarization properties of ASKAP-localized fast radio bursts. *Mon. Not. R. Astron. Soc.* **497**, 3335–3350 (2020).

31. E. Fonseca, B. C. Andersen, M. Bhardwaj, P. Chawla, D. C. Good, A. Josephy, V. M. Kaspi, K. W. Masui, R. Mckinven, D. Michilli, Z. Pleunis, K. Shin, S. P. Tendulkar, K. M. Bandura, P. J. Boyle, C. Brar, T. Cassanelli, D. Cubranic, M. Dobbs, F. Q. Dong, B. M. Gaensler, G. Hinshaw, T. L. Landecker, C. Leung, D. Z. Li, H. H. Lin, J. Mena-Parra, M. Merryfield, A. Naidu, C. Ng, C. Patel, U. Pen, M. Rafiei-Ravandi, M. Rahman, S. M. Ransom, P. Scholz, K. M. Smith, I. H. Stairs, K. Vanderlinde, P. Yadav, A. V. Zwaniga, Nine new repeating fast radio burst sources from CHIME/FRB. *Astrophys. J.* **891**, L6 (2020).

32. M. Pick, N. Vilmer, Sixty-five years of solar radioastronomy: Flares, coronal mass ejections and Sun Earth connection. *Astron. Astrophys. Rev.* **16**, 1–153 (2008).

33. H. A. S. Reid, H. Ratcliffe, A review of solar type III radio bursts. *Res. Astron. Astrophys.* **14**, 773–804 (2014).

34. E. Platts, M. Caleb, B. W. Stappers, R. A. Main, A. Weltman, J. P. Shock, M. Kramer, M. C. Bezuidenhout, F. Jankowski, V. Morello, A. Possenti, K. M. Rajwade, L. Rhodes, J. Wu, An analysis of the time-frequency structure of several bursts from FRB 121102 detected with MeerKAT. *Mon. Not. R. Astron. Soc.* **505**, 3041–3053 (2021).

35. D. J. Zhou, J. L. Han, B. Zhang, K. J. Lee, W. W. Zhu, D. Li, W. C. Jing, W. Y. Wang, Y. K. Zhang, J. C. Jiang, J. R. Niu, R. Luo, H. Xu, C. F. Zhang, B. J. Wang, J. W. Xu, P. Wang, Z. L. Yang, Y. Feng, FAST observations of an extremely active episode of FRB 20201124A: I. Burst morphology. *Res. Astron. Astrophys.* **22**, 124001 (2022).

36. J. N. Jahns, L. G. Spitler, K. Nimmo, D. M. Hewitt, M. P. Snelders, A. Seymour, J. W. T. Hessels, K. Gourdji, D. Michilli, G. H. Hilmarsson, The FRB 20121102A November rain in 2018 observed with the Arecibo Telescope. *Mon. Not. R. Astron. Soc.* **519**, 666–687 (2023).

37. G. H. Hilmarsson, L. G. Spitler, R. A. Main, D. Z. Li, Polarization properties of FRB 20201124A from detections with the Effelsberg 100-m radio telescope. *Mon. Not. R. Astron. Soc.* **508**, 5354–5361 (2021).

38. Z. Pleunis, D. C. Good, V. M. Kaspi, R. Mckinven, S. M. Ransom, P. Scholz, K. Bandura, M. Bhardwaj, P. J. Boyle, C. Brar, T. Cassanelli, P. Chawla, F. Dong, E. Fonseca, B. M. Gaensler, A. Josephy, J. F. Kaczmarek, C. Leung, H.-H. Lin, K. W. Masui, J. Mena-Parra, D. Michilli, C. Ng, C. Patel, M. Rafiei-Ravandi, M. Rahman, P. Sanghavi, K. Shin, K. M. Smith, I. H. Stairs, S. P. Tendulkar, Fast radio burst morphology in the first CHIME/FRB catalog. *Astrophys. J.* **923**, 1 (2021).

39. W. Wang, B. Zhang, X. Chen, R. Xu, On the time-frequency downward drifting of repeating fast radio bursts. *Astrophys. J.* **876**, L15 (2019).

40. M. Lyutikov, Radius-to-frequency mapping and FRB frequency drifts. *Astrophys. J.* **889**, 135 (2020).

41. B. D. Metzger, N. Sridhar, B. Margalit, P. Beniamini, L. Sironi, A toy model for the time-frequency structure of fast radio bursts: Implications for the CHIME/FRB burst dichotomy. *Astrophys. J.* **925**, 135 (2022).

42. E. Kundu, B. Zhang, Free-free absorption in hot relativistic flows: Application to fast radio bursts. *Mon. Not. R. Astron. Soc.* **508**, L48–L52 (2021).

43. J. E. Everett, J. M. Weisberg, Emission beam geometry of selected pulsars derived from average pulse polarization data. *Astrophys. J.* **553**, 341–357 (2001).

44. V. Radhakrishnan, J. M. Rankin, Toward an empirical theory of pulsar emission. V. On the circular polarization in pulsar radiation. *Astrophys. J.* **352**, 258 (1990).

45. M. Kramer, B. W. Stappers, A. Jessner, A. G. Lyne, C. A. Jordan, Polarized radio emission from a magnetar. *Mon. Not. R. Astron. Soc.* **377**, 107–119 (2007).

46. S. Dai, M. E. Lower, M. Bailes, F. Camilo, J. P. Halpern, S. Johnston, M. Kerr, J. Reynolds, J. Sarkissian, P. Scholz, Wideband polarized radio emission from the newly revived magnetar XTE J1810-197. *Astrophys. J.* **874**, L14 (2019).

47. H. Tong, P. F. Wang, H. G. Wang, Z. Yan, Rotating vector model for magnetars. *Mon. Not. R. Astron. Soc.* **502**, 1549–1556 (2021).

48. R. Luo, B. J. Wang, Y. P. Men, C. F. Zhang, J. C. Jiang, H. Xu, W. Y. Wang, K. J. Lee, J. L. Han, B. Zhang, R. N. Caballero, M. Z. Chen, X. L. Chen, H. Q. Gan, Y. J. Guo, L. F. Hao, Y. X. Huang, P. Jiang, H. Li, J. Li, Z. X. Li, J. T. Luo, J. Pan, X. Pei, L. Qian, J. H. Sun, M. Wang, N. Wang, Z. G. Wen, R. X. Xu, Y. H. Xu, J. Yan, W. M. Yan, D. J. Yu, J. P. Yuan, S. B. Zhang, Y. Zhu, Diverse polarization angle swings from a repeating fast radio burst source. *Nature* **586**, 693–696 (2020).

49. S. Dai, G. Hobbs, R. N. Manchester, M. Kerr, R. M. Shannon, W. van Straten, A. Mata, M. Bailes, N. D. R. Bhat, S. Burke-Spolaor, W. A. Coles, S. Johnston, M. J. Keith, Y. Levin, S. Osłowski, D. Reardon, V. Ravi, J. M. Sarkissian, C. Tiburzi, L. Toomey, H. G. Wang, J. B. Wang, L. Wen, R. X. Xu, W. M. Yan, X. J. Zhu, A study of multifrequency polarization pulse profiles of millisecond pulsars. *Mon. Not. R. Astron. Soc.* **449**, 3223–3262 (2015).

50. H. Xu, J. R. Niu, P. Chen, K. J. Lee, W. W. Zhu, S. Dong, B. Zhang, J. C. Jiang, B. J. Wang, J. W. Xu, C. F. Zhang, H. Fu, A. V. Filippenko, E. W. Peng, D. J. Zhou, Y. K. Zhang, P. Wang, Y. Feng, Y. Li, T. G. Brink, D. Z. Li, W. Lu, Y. P. Yang, R. N. Caballero, C. Cai, M. Z. Chen, Z. G. Dai, S. G. Djorgovski, A. Esamdin, H. Q. Gan, P. Guhathakurta, J. L. Han, L. F. Hao, Y. X. Huang, P. Jiang, C. K. Li, D. Li, H. Li, X. Q. Li, Z. X. Li, Z. Y. Liu, R. Luo, Y. P. Men, C. H. Niu, W. X. Peng, L. Qian, L. M. Song, D. Stern, A. Stockton, J. H. Sun, F. Y. Wang, M. Wang, N. Wang, W. Y. Wang, X. F. Wu, S. Xiao, S. L. Xiong, Y. H. Xu, R. X. Xu, J. Yang, X. Yang, R. Yao, Q. B. Yi, Y. L. Yue, D. J. Yu, W. F. Yu, J. P. Yuan, B. B. Zhang, S. B. Zhang, S. N. Zhang, Y. Zhao, W. K. Zheng, Y. Zhu, J. H. Zou, A fast radio burst source at a complex magnetized site in a barred galaxy. *Nature* **609**, 685–688 (2022).

51. J.-C. Jiang, W.-Y. Wang, H. Xu, J.-W. Xu, C.-F. Zhang, B.-J. Wang, D.-J. Zhou, Y.-K. Zhang, J.-R. Niu, K.-J. Lee, B. Zhang, J.-L. Han, D. Li, W.-W. Zhu, Z.-G. Dai, Y. Feng, W.-C. Jing, D.-Z. Li, R. Luo, C.-C. Miao, C.-H. Niu, C.-W. Tsai, F.-Y. Wang, P. Wang, R.-X. Xu, Y.-P. Yang, Z.-L. Yang, J.-M. Yao, M. Yuan, FAST observations of an extremely active episode of FRB 20201124A. III. Polarimetry. *Res. Astron. Astrophys.* **22**, 124003 (2022).

52. M. E. Lower, A phenomenological model for measuring generalised Faraday rotation. arXiv:2108.09429 [astro-ph.HE] (2021).

53. M. E. Lower, S. Johnston, M. Lyutikov, D. B. Melrose, R. M. Shannon, P. Weltevrede, M. Caleb, F. Camilo, A. D. Cameron, S. Dai, G. Hobbs, D. Li, K. M. Rajwade, J. E. Reynolds, J. M. Sarkissian, B. W. Stappers, Linear to circular conversion in the polarized radio emission of a magnetar. *Nat. Astron.* **8**, 606–616 (2024).

54. P. Kumar, R. M. Shannon, M. E. Lower, A. T. Deller, J. X. Prochaska, Propagation of a fast radio burst through a birefringent relativistic plasma. *Phys. Rev. D* **108**, 043009 (2023).

55. A. Gruzinov, Y. Levin, Conversion measure of faraday rotation-conversion with application to fast radio bursts. *Astrophys. J.* **876**, 74 (2019).

56. Jonas & MeerKAT Team 2016, MeerKAT Science: On the Pathway to the SKA, Proceedings of Science 1 10.22323/1.277.0001.

57. M. Bailes, A. Jameson, F. Abbate, E. D. Barr, N. D. R. Bhat, L. Bondonneau, M. Burgay, S. J. Buchner, F. Camilo, D. J. Champion, I. Cognard, P. B. Demorest, P. C. C. Freire, T. Gautam, M. Geyer, J. M. Griessmeier, L. Guillemot, H. Hu, F. Jankowski, S. Johnston, A. Karastergiou, R. Karuppusamy, D. Kaur, M. J. Keith, M. Kramer, J. van Leeuwen, M. E. Lower, Y. Maan, M. A. McLaughlin, B. W. Meyers, S. Osłowski, L. S. Oswald, A. Parthasarathy, T. Pennucci, B. Posselt, A. Possenti, S. M. Ransom, D. J. Reardon, A. Ridol i, C. T. G. Schollar, M. Serylak, G. Shaifullah, M. Shamohammadi, R. M. Shannon, C. Sobey, X. Song, R. Spiewak, I. H. Stairs, B. W. Stappers, W. van Straten, A. Szary, G. Theureau, V. V. Krishnan, P. Weltevrede, N. Wex, T. D. Abbott, G. B. Adams, J. P. Burger, R. R. G. Gamatham, M. Gouws, D. M. Horn, B. Hugo, A. F. Joubert, J. R. Manley, K. McAlpine, S. S. Passmoor, A. Peens-Hough, Z. R. Ramudzuli, A. Rust, S. Salie, L. C. Schwardt, R. Siebrits,

G. Van Tonder, V. Van Tonder, M. G. Welz, The MeerKAT telescope as a pulsar facility: System verification and early science results from MeerTime. *Astron. Soc. Aust.* **37**, e028 (2020).

58. M. Serylak, S. Johnston, M. Kramer, S. Buchner, A. Karastergiou, M. J. Keith, A. Parthasarathy, P. Weltevrede, M. Bailes, E. D. Barr, F. Camilo, M. Geyer, B. V. Hugo, A. Jameson, D. J. Reardon, R. M. Shannon, R. Spiewak, W. van Straten, V. Venkatraman Krishnan, The thousand-pulsar-array programme on MeerKAT IV: Polarization properties of young, energetic pulsars. *Mon. Not. R. Astron. Soc.* **505**, 4483–4495 (2021).

59. W. van Straten, M. Bailes, DSPSR: Digital signal processing software for pulsar astronomy. *Astron. Soc. Aust.* **28**, 1–14 (2011).

60. Y. Men, E. Barr, TransientX: A high performance single pulse search package. arXiv:2401.13834 [astro-ph.IM] (2024).

61. A. Seymour, D. Michilli, Z. Pleunis, DM_phase: Algorithm for correcting dispersion of radio signals, Astrophysics Source Code Library, record ascl:1910.004 (2019).

62. C. Sotomayor-Beltran, C. Sobey, J. W. T. Hessels, G. de Bruyn, A. Noutsos, A. Alexov, J. Anderson, A. Asgekar, I. M. Avruch, R. Beck, M. E. Bell, M. R. Bell, M. J. Bentum, G. Bernardi, P. Best, L. Birzan, A. Bonafede, F. Breitling, J. Broderick, W. N. Brouw, M. Brüggen, B. Ciardi, F. de Gasperin, R. J. Dettmar, A. van Duin, S. Duscha, J. Eislöffel, H. Falcke, R. A. Fallows, R. Fender, C. Ferrari, W. Frieswijk, M. A. Garrett, J. Grießmeier, T. Grit, A. W. Gunst, T. E. Hassall, G. Heald, M. Hoeft, A. Horneffer, M. Iacobelli, E. Juette, A. Karastergiou, E. Keane, J. Kohler, M. Kramer, V. I. Kondratiev, L. V. E. Koopmans, M. Kuniyoshi, G. Kuper, J. van Leeuwen, P. Maat, G. Macario, S. Markoff, J. P. McKean, D. D. Mulcahy, H. Munk, E. Orru, H. Paas, M. Pandey-Pommier, M. Pilia, R. Pizzo, A. G. Polatidis, W. Reich, H. Röttgering, M. Serylak, J. Sluman, B. W. Stappers, M. Tagger, Y. Tang, C. Tasse, S. ter Veen, R. Vermeulen, R. J. van Weeren, R. A. M. J. Wijers, S. J. Wijnholds, M. W. Wise, O. Wucknitz, S. Yatawatta, P. Zarka, Calibrating high-precision Faraday rotation measurements for LOFAR and the next generation of low-frequency radio telescopes. *Astron. Astrophys* **552**, A58 (2013).

63. J. M. Yao, R. N. Manchester, N. Wang, A new electron-density model for estimation of pulsar and FRB distances. *Astrophys. J.* **835**, 29 (2017).

64. A. A. Nowroozi, Table for fisher's test of significance in harmonic analysis. *Geophys. J. Int.* **12**, 517–520 (1967).

65. A. Savitzky, M. J. E. Golay, Smoothing and differentiation of data by simplified least squares procedures. *Anal. Chem.* **36**, 1627–1639 (1964).

66. F. Feroz, M. P. Hobson, Multimodal nested sampling: An efficient and robust alternative to Markov Chain Monte Carlo methods for astronomical data analyses. *Mon. Not. R. Astron. Soc.* **384**, 449–463 (2008).

67. J. Skilling, *Bayesian Inference and Maximum Entropy Methods in Science and Engineering: 24th International Workshop on Bayesian Inference and Maximum Entropy Methods in Science and Engineering*, R. Fischer, R. Preuss, U. V. Toussaint, Eds. (American Institute of Physics Conference Series, 2004), vol. 735, pp. 395–405.

68. M. J. Keith, S. Johnston, A. Karastergiou, P. Weltevrede, M. E. Lower, A. Basu, B. Posselt, L. S. Oswald, A. Parthasarathy, A. D. Cameron, M. Serylak, S. Buchner, The Thousand-Pulsar-Array programme on MeerKAT - XIII. Timing, flux density, rotation measure, and dispersion measure time series of 597 pulsars. *Mon. Not. R. Astron. Soc.* **530**, 1581–1591 (2024).

69. S. A. Petrova, On the origin of orthogonal polarization modes in pulsar radio emission. *Astron. Astrophys* **378**, 883–897 (2001).

70. R. N. Manchester, J. H. Taylor, G. R. Huguenin, Observations of pulsar radio emission. II. Polarization of individual pulses. *Astrophys. J.* **196**, 83–102 (1975).

71. D. B. Melrose, Propagation effects on the polarization of pulsar radio emission. *Aust. J. Phys.* **32**, 61–70 (1979).

72. Y. Qu, B. Zhang, Polarization of fast radio bursts: Radiation mechanisms and propagation effects. *Mon. Not. R. Astron. Soc.* **522**, 2448–2477 (2023).

73. G. J. Qiao, W. P. Lin, An inverse Compton scattering (ICS) model of pulsar emission. I. Core and conal emission beams. *Astron. Astrophys* **333**, 172–180 (1998).

74. Y. Qu, B. Zhang, Coherent inverse compton scattering in fast radio bursts revisited. *Astrophys. J.* **972**, 124 (2024).

75. M. Kennett, D. Melrose, Propagation-induced circular polarisation in synchrotron sources. *Astron. Soc. Aust.* **15**, 211–216 (1998).

76. M. Lyutikov, Faraday conversion in pair-symmetric winds of magnetars and fast radio bursts. *Astrophys. J.* **933**, L6 (2022).

77. H. K. Vedantham, V. Ravi, Faraday conversion and magneto-ionic variations in fast radio bursts. *Mon. Not. R. Astron. Soc.* **485**, L78–L82 (2019).

78. A. F. Cheng, M. A. Ruderman, A theory of subpulse polarization patterns from radio pulsars. *Astrophys. J.* **229**, 348–360 (1979).

79. P. Beniamini, P. Kumar, R. Narayan, Faraday depolarization and induced circular polarization by multipath propagation with application to FRBs. *Mon. Not. R. Astron. Soc.* **510**, 4654–4668 (2022).

80. Chime/Frb Collaboration, B. C. Andersen, K. Bandura, M. Bhardwaj, P. J. Boyle, C. Brar, T. Cassanelli, S. Chatterjee, P. Chawla, A. M. Cook, A. P. Curtin, M. Dobbs, F. A. Dong, J. T. Faber, M. Fandino, E. Fonseca, B. M. Gaensler, U. Giri, A. Herrera-Martin, A. S. Hill, A. Ibik, A. Josephy, J. F. Kaczmarek, Z. Kader, V. Kaspi, T. L. Landecker, A. E. Lanman, M. Lazda, C. Leung, H.-H. Lin, K. W. Masui, R. McKinven, J. Mena-Parra, B. W. Meyers, D. Michilli, C. Ng, A. Pandhi, A. B. Pearlman, U.-L. Pen, E. Petroff, Z. Pleunis, M. Rafiei-Ravandi, M. Rahman, S. M. Ransom, A. Renard, K. R. Sand, P. Sanghavi, P. Scholz, V. Shah, K. Shin, S. Siegel, K. Smith, I. Stairs, J. Su, S. P. Tendulkar, K. Vanderlinde, H. Wang, D. Wulf, A. Zwaniga, CHIME/FRB discovery of 25 repeating fast radio burst sources. *Astrophys. J.* **947**, 83 (2023).
